# Viremia and nasal shedding for the diagnosis of equine herpesvirus‐1 infection in domesticated horses

**DOI:** 10.1111/jvim.16958

**Published:** 2023-12-09

**Authors:** Nicola Pusterla, David C. Dorman, Brandy A. Burgess, Lutz Goehring, Margaret Gross, Klaus Osterrieder, Gisela Soboll Hussey, David P. Lunn

**Affiliations:** ^1^ School of Veterinary Medicine University of California Davis California USA; ^2^ College of Veterinary Medicine North Carolina State University Raleigh North Carolina USA; ^3^ College of Veterinary Medicine University of Georgia Athens Georgia USA; ^4^ College of Agriculture, Food and Environment, Maxwell H. Gluck Equine Research Center University of Kentucky Lexington Kentucky USA; ^5^ Institut für Virologie, Freie Universität Berlin Berlin Germany; ^6^ College of Veterinary Medicine Michigan State University, Veterinary Medical Center East Lansing Michigan USA; ^7^ School of Veterinary Science University of Liverpool, Leahurst Campus Neston UK

**Keywords:** abortion, equine, equine herpesvirus myeloencephalopathy, herpesvirus‐1, nasal shedding, quantitative polymerase chain reaction (qPCR), rhinopneumonitis, viremia, virus isolation

## Abstract

**Background:**

Equine herpesvirus type 1 (EHV‐1) infection is associated with upper respiratory disease, EHM, abortions, and neonatal death.

**Research Questions:**

Are nasal secretions a more sensitive biological sample compared to blood for the detection of EHV‐1 infection? How long is EHV‐1 detectable after primary infection by PCR?

**Methods:**

MedLine and Web of Science searches identified original peer‐reviewed reports evaluating nasal shedding and viremia using virus isolation methods or PCR published in English before October 9, 2023.

**Results:**

Sixty experimental and 20 observational studies met inclusion criteria. EHV‐1 detection frequency by qPCR in nasal secretions and blood from naturally‐infected horses with fever and respiratory signs were 15% and 9%, respectively; qPCR detection rates in nasal secretions and blood from horses with suspected EHM were 94% and 70%, respectively. In experimental studies the sensitivity of qPCR matched or exceeded that seen for virus isolation from either nasal secretions or blood. Detection of nasal shedding typically occurred within 2 days after EHV‐1 inoculation with a detection period of 3 to 7 days. Viremia lasted 2 to 7 days and was usually detected ≥1 days after positive identification of EHV‐1 in nasal secretions. Nasal shedding and viremia decreased over time and remained detectable in some horses for several weeks after inoculation.

**Conclusions and Clinical Importance:**

Under experimental conditions, blood and nasal secretions have similar sensitivity for the detection of EHV‐1 when horses are sampled on multiple consecutive days. In contrast, in observational studies detection of EHV‐1 in nasal secretions was consistently more successful.

**Abbreviations**
EHM: equine herpesvirus‐1 myeloencephalopathy
EHV‐1: equine herpesvirus‐1
GRADE: Grading of Recommendations, Assessment, Development, and Evaluation
PICO: population, intervention, comparator, and outcome
RCTs: randomized clinical trials

## 1 INTRODUCTION

Equine herpesvirus 1 (EHV‐1) is a highly contagious alpha‐herpesvirus that affects equids worldwide. Horse‐to‐horse transmission is frequently the result of direct contact with nasal discharge, or aborted fetuses, while indirect contact can also occur through fomites.^1^

Virus replication in the upper respiratory tract results in local inflammation, which is associated with fever, lethargy, anorexia, nasal discharge, coughing and mandibular lymphadenopathy. EHV‐1 infects the respiratory epithelium from which it is transported to regional lymph nodes before establishing a cell‐associated viremia which is a prerequisite for the most serious sequelae of infection, which are equine herpesvirus myeloencephalopathy (EHM) or abortion. Experimentally, EHV‐1 shedding is reported to occur from the respiratory tract for up to 14 days, while viremia can persist for up to 21 days.^1^

Diagnosis of EHV‐1 infection cannot be made solely based on clinical signs. Definitive diagnosis of EHV‐1 infection can be determined by inoculation of nasal secretions (swabs) and/or peripheral mononuclear cells (PBMC) isolated from blood in cell cultures for the isolation of the virus. EHV‐1 can be isolated in a variety of cell lines including equine kidney (EK), equine fibroblast (NBL‐6 or E‐Derm), rabbit kidney (RK‐13), or Madin‐Darby bovine kidney (MDBK) cells.^2^ Infection of cells with EHV‐1 results in a cytopathic effect (CPE) characterized by enlarged, rounded, and ultimately detached cells. Molecular diagnosis of EHV‐1 infection using conventional PCR added significantly to the speed of virus detection, but in the past 2 decades the use of quantitative real time polymerase chain reaction (qPCR) techniques has supplanted conventional detection methods including cell culture and conventional PCR because of its high sensitivity, specificity, quick turn‐around‐time, and cost‐effectiveness.^3^ Nasal secretions alone or in combination with blood are often used for the qPCR detection of EHV‐1 in clinically affected horses. Disadvantages of PCR include the need for highly trained laboratory staff, risk of contamination, and potential inability to detect novel DNA sequences.

The goal of this study was to summarize and review the available literature to address the following research questions: (a) Are nasal secretions a more sensitive biological sample compared to blood for the detection of EHV‐1 infection; and (b), how long is EHV‐1 detectable after primary infection by PCR?

## 2 METHODS

This review did not involve animal data collection. Therefore, ethical approval was not required. A PICO (problem/population, intervention, comparison, and outcome) framework was developed to help guide this review:

- Population: Domesticated horses (*Equus caballus*) without sex, age, or breed restrictions.
- Intervention/Exposure: Horses experimentally infected or naturally exposed to EHV‐1 infection.
- Comparator: Measurement/detection of duration of EHV‐1 viremia and of nasal shedding after infection in the same horses.
- Outcome: Presence and duration of viremia and nasal shedding of EHV‐1 over time postinfection.

Studies were included if they had the following features:

- Domesticated equids without age, breed, or immunological status restriction
- Any experimental challenge or natural infection with subsequent measurement of nasal shedding and viremia.
- Peer‐reviewed original data.
- Published in English.
- Study included clinical outcomes that reflect clinical EHV‐1 infection resulting in either pyrexia, respiratory signs, abortion, neonatal loss, or EHM.

### 2.1 Search strategy

A search for bibliographic references was performed with the assistance of a librarian to locate studies that evaluated nasal shedding and viremia in horses following EHV‐1 infection. A systematic literature search was performed in PubMed. The search was adapted for Web of Science, Agricola, and Global Index Medicus Regional Databases. The search was limited to domesticated horses and was performed without sex, age, or breed restrictions. Search terms are available in Supplementary Materials Item 1. Searches were last performed on 10/9/2023.

### 2.2 Study selection

Screening was tracked in Covidence (www.covidence.org). The evaluation of titles, abstracts, and the full text were independently performed by teams of 2 reviewers; disagreements were resolved by either discussion or when consensus could not be reached using a third reviewer.

### 2.3 Data extraction

Data were extracted from included studies by 1 member of the review team. Extraction of graphical data relied on DigitizeIt version 2.5.1 (Braunschweig, Germany). Extracted data were used to summarize study designs and findings and/or to conduct post hoc statistical analyses. Extracted data included: demographic data, challenge infection protocol including virus strain and dose, incidence of pyrexia, abortion, or neurologic signs, and virological data including the presence or absence of either nasal shedding or viremia considering the duration, quantitation, and methods used.

### 2.4 Data analysis

Incidence data were used to calculate the sensitivity of virus isolation and PCR as diagnostic tests for the detection of EHV‐1 in either nasal swabs or nasopharyngeal swabs (collectively referred to as nasal swabs) or from whole blood or purified PBMCs. For each comparison, we extracted data on the number of true positives, true negatives, false positives, and false negatives in the form of a 2‐by‐2 table. In some cases, studies had multiple arms (eg, different virus strains, doses, age groups). Data were pooled when results across the different arms yielded identical results. Analyses evaluating test sensitivity were performed on experimental datasets from studies with known exposure to EHV‐1; thus, specificity could not be determined. Unless otherwise noted, means ± standard deviations are provided. Statistical calculations were performed using MedCalc Software Ltd (Ostend, Belgium).

## 3 RESULTS

### 3.1 Results of the search

**FIGURE 1:**
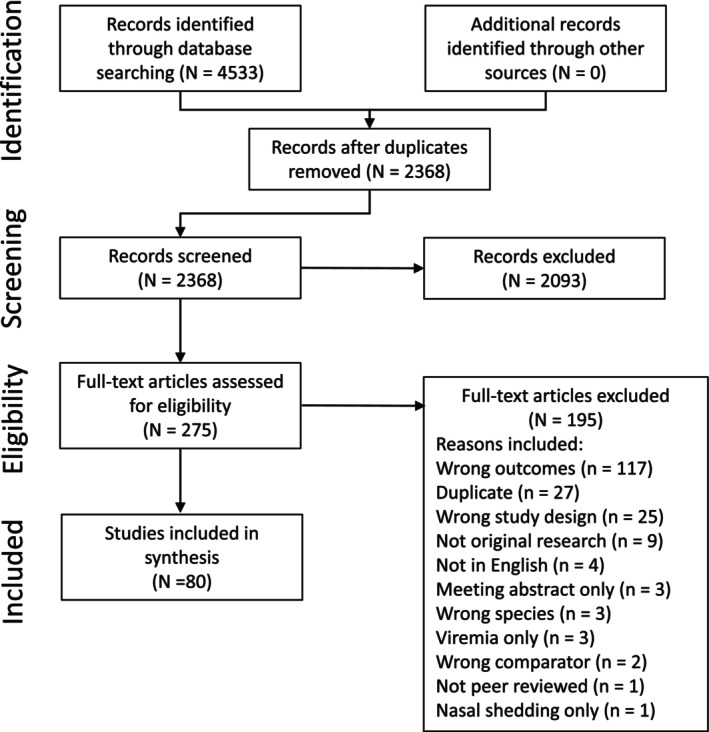
PRISMA flowchart for the literature search process.

The search identified 4533 citations, of which 2165 were duplicate citations. Another 2095 citations were excluded based on a review of the title and abstract. Literature was almost entirely identified and retrieved from electronic bibliographic sources. No studies were identified from hand searching reference lists provided in the studies that met inclusion criteria. A total of 275 studies were assessed for inclusion using a review of the full text. A list of the 195 studies excluded at the full text review stage, with the reason for exclusion, are provided in Supplementary Materials Item 2. Eighty studies met the inclusion criteria for this review, and a flow diagram for inclusion of studies is provided in Figure 1.

### 3.2 Description of the included observational studies

A total of 20 observational studies met our inclusion criteria.^4^, ^5^, ^6^, ^7^, ^8^, ^9^, ^10^, ^11^, ^12^, ^13^, ^14^, ^15^, ^16^, ^17^, ^18^, ^19^, ^20^, ^21^, ^22^, ^23^ The key characteristics of these studies are reported in Table 1. Two studies used virus isolation methods to detect EHV‐1.^6^, ^23^ All studies used PCR either as the only method or in combination with virus isolation, and the large majority (14 of 20) used qPCR (Table 1). Several of the observational studies evaluated nasal shedding and viremia in horses with fever or cough and other respiratory signs consistent with EHV‐1 infection.^4^, ^6^, ^10^, ^13^, ^15^, ^16^, ^18^, ^19^, ^20^, ^22^, ^23^ Other studies performed diagnostic testing in horses with either neurologic signs consistent with EHM or episodic abortions.^5^, ^6^, ^7^, ^9^, ^11^, ^12^, ^13^, ^14^, ^15^, ^16^, ^17^, ^18^, ^20^, ^21^ For all horses in the studies presenting with fever and respiratory signs for which clinical and laboratory information was available, the detection of EHV‐1 by PCR in nasal secretions and blood was 15% and 9%, respectively.^4^, ^6^, ^10^, ^13^, ^15^, ^16^, ^18^, ^19^, ^20^, ^22^, ^23^ For the same study populations, the detection of EHV‐1 in nasal secretions and blood by culture was 2.7% and 0.2%, respectively. For horses with EHM, the PCR detection of EHV‐1 in nasal secretions and blood was 94% and 70%, respectively.^5^, ^6^, ^7^, ^9^, ^11^, ^12^, ^13^, ^14^, ^15^, ^16^, ^17^, ^18^, ^20^, ^21^ In mares that aborted their foals, EHV‐1 was detected by PCR in 30% of nasal secretions and 37% of blood samples.^6^, ^17^, ^18^, ^20^, ^21^ Duration of shedding of EHV‐1 during an outbreak can be variable. During of abortion outbreaks can be extensive, with EHV‐1 detection in nasal swabs using qPCR methods occurring throughout the course of the outbreak as horses become infected.^6^ Similarly, in outbreaks not involving abortion serial sampling of individual horses and analysis of nasal swabs using qPCR showed the presence of EHV‐1 DNA in samples for as long as 42 days during an outbreak.^20^

**TABLE 1:** Main outcomes from observational studies where both nasal samples and blood were evaluated.

Study	Clinical outcomes	Virus isolation detection method	Nasal shedding	Viremia	Comments
Ataseven et al., 2009^4^	Pneumonia or upper respiratory disease (incidence: 21/21)	Multiplex nested PCR	Overall (+): 9/21 horses	Overall (+): 3/21 horses	Study also screened for EHV‐4
Brown et al., 2007^5^	Farm with prior abortion outbreaks	RT qPCR	Overall (+): 3/590 horses	Overall (+): 0/590 horses	
Damiani et al., 2014^6^	Abortion outbreak with an incidence of 16/25. Fever: 4/25. Weak foals: 2/25. Ataxia: 1/25	Plaque assay RK13 cells	Incidence @ day 16:1/23. Peak incidence (day 21): 2/21	Overall (+): @ day 16:1/22; ***day 23***:1/21	Paired blood and nasal swab samples were only collected on day 16 of the outbreak. Some animals did not have samples collected at all timepoints
		RT qPCR	Overall (+) @ day 16:3/23, @ day 21:6/21, @ ***day 72***:3/4 horses	Overall (+): @ day 16:7/22, ***day 23***:1/21 horses	
Estell et al., 2015^7^	EHM: 7/7. Case fatality: 2/7	RT qPCR	Peak viral load ranged from 6.9 × 10^3^ to 2.81 × 10^5^ gB gene copies/10^6^ cells. Median: 5.11 × 10^4^ gB gene copies/10^6^ cells. Detectable shedding ceased on day 5	Peak viral load ranged from 143 to 4340 gB gene copies/10^6^ cells. Median: 3150 gB gene copies/10^6^ cells. Detectable viremia ceased on day 6	Survivors (n = 5)
			Peak viral load: 1.9 × 10^9^ and 2.2 × 10^9^ gB gene copies/10^6^ cells	Peak viral load: 2.05 × 10^4^ and 1.02 × 10^5^ gB gene copies/10^6^ cells	Nonsurvivors (n = 2)
Marenzoni et al., 2008^8^	Foals (n = 15) were sequentially sampled and screened for EHV‐1	Semi nested PCR	Overall (+): 20/60 samples	Overall (+): 54/60 samples	Study also screened for EHV‐4. Each foal was screened approximately every 6 weeks from 1 to 6 mo of age (n = 4 samples/foal)
McFadden et al., 2016^9^	EHM (n = 15)	RT qPCR	Overall (+) 1st sample: 6/8 horses 29.7 ± 5.0 Cq (n = 6) Overall (+) 2nd sample @ day 25 to 37:5/6 horses (Cq = 38.3 @ day 25)	Overall (+) 1st sample: 7/9 horses 33.9 ± 2.8 Cq (n = 7) Blood or buffy coat Overall (+) 2nd sample @ day 37:0/7 horses	Some horses were not sampled. Cq cut off <38
Ohta et al., 2011^10^	Fever (rectal temperature >38.5°C)	PCR	Overall (+): 19/124 horses	Overall (+): 22/124 horses	EHV‐1 DNA was also detected in horses without seroconversion at a low rate
Pronost et al., 2012^11^	EHM: 7/66, Fever: 6/7	RT qPCR	Overall (+): 4/4 horses 32.1 ± 5.7 Ct (n = 4)	Overall (+): 6/6 horses 34.5 ± 2.0 Ct (n = 6)	Some missing samples reported. Affected riding school had 66 horses
Pusterla et al., 2008^12^	Surveillance at a racetrack with a previous EHM outbreak (n = 146)	RT qPCR	Nasal swab: Overall (+) 28/118 horses. gB gene: 32.87 ± 4.78 Ct NP swab: Overall (+) 24/122 horses, gB gene: 32.1 ± 5.81 Ct	ND	Racetrack horses were not febrile or displaying neurological signs at the time of collection of a nasal swab and nasopharyngeal swab
Pusterla et al., 2008^13^	Acute onset of fever (n = 12)	RT qPCR	Overall (+) 12/12 horses. Viral load: 2800 ± 2500 gene copies/10^6^ cells	Overall (+) 12/12 horses. Viral load: 17000 ± 9700 gene copies/10^6^ cells	Febrile horses had higher viral loads in blood (*P* 0.001) than neurological and subclinical horses. Viral loads in nasal secretions of neurological horses were higher than those of febrile horses or subclinically infected horses (*P* 0.001)
	Neurologic signs (n = 15)		Overall (+) 15/15 horses. Viral load: 1.0 × 10^6^ gene copies/10^6^ cells	Overall (+) 9/15 horses. Viral load: 160 gene copies/10^6^ cells	Febrile horses had higher viral loads in blood (p 0.001) than neurological and subclinical horses. Viral loads in nasal secretions of neurological horses were higher than those of febrile horses or subclinically infected horses (p 0.001) Healthy adult horses with a history of recent transportation (n = 302) of which four tested positive for EHV‐1. Horse with highest nasal viral load developed fever
	Subclinical (n = 41)		Overall (+) 40/41 horses. Viral load: 8600 gene copies/10^6^ cells	Overall (+) 5/41 horses. Viral load: 45 gene copies/10^6^ cells	
	Healthy adult horses (n = 3). Fever (n = 1)		Overall (+): 3/4 horses. Viral load: 200, 3000, and 2 × 10^7^ gene copies/10^6^ cells. PCR (+) sample from horse with highest viral load was present until day 11	Overall (+): 2/4 horses. Viral load: 100 and 6000 gene copies/10^6^ cells	
Pusterla et al., 2009^14^	Surveillance at a racetrack with a previous EHM outbreak (n = 74)	RT qPCR	Overall (+): 14/14 horses. Viral load range: 62 to 1.9 × 10^8^ gene copies/10^6^ cells	Overall (+): 4/14 horses. Viral load range: 180 to 4.9 × 10^3^ gene copies/10^6^ cells	Data provided for first test date (n = 14 horses that tested PCR+)
Pusterla et al. 2009^15^	Surveillance at a racetrack with a previous EHM outbreak (n = 74) Upper respiratory tract infection and/or neurological deficits (n = 761 horses)	RT qPCR (gDNA)	Overall (+): 6/14 horses. Viral load range: 89 to 2.1 × 10^7^ gene copies/10^6^ cells. 2/14 horses were PCR (+) on day 22.	Overall (+): 2/14 horses. Viral load range: 1600 to 4.8 × 10^4^ gene copies/10^6^ cells.	Data provided for first test date (n = 14 horses that tested PCR+) 761 horses enrolled. PCR (+) for both outcomes: 6/761. Data included in individual outcome data
		RT qPCR (cDNA)	Overall (+): 21/761 horses	Overall (+): 8/761 horses	
Pusterla et al., 2011^16^	Upper respiratory tract infection and/or neurological deficits (n = 4228 horses)	RT qPCR	Overall (+): 100/117 horses	Overall (+): 47/117 horses	Data from 117 cases that tested qPCR‐positive for both the gB and ORF 30 EHV‐1 genes, of these 33 horses had positive results for both samples
Pusterla et al., 2016^17^	Abortion and/or EHM (n = 10)	RT qPCR	Overall (+): 1/10 horses	Overall (+): 7/10 horses	Two foals also had nasal swabs = (+)
Pusterla et al., 2021^18^	Upper respiratory tract infection (n = 26) and/or EHM (n = 4)	RT qPCR	Overall (+): 26/31 horses	Overall (+): 18/31 horses	First study in the USA documenting H_752_ EHV‐1 genotype
Pusterla et al., 2023^19^	Acute onset of fever and upper respiratory tract infection	RT qPCR	Overall (+): 13/667 equids	Overall (+):4/667 equids	Population includes <2% donkeys and mules
Studdert et al., 2003^20^	Outbreaks of respiratory disease (n = 23), abortions (n = 4), and EHM (n = 2)	Semi nested PCR	Overall (+) @ day 14:8/14 horses. Two horses remained positive until day 42	Overall (+) @ day 14:6/14 horses. One horse remained positive until day 35	Data from 15 horses with weekly sampling. Paired nasal and blood samples collected starting on day 14 and ending on day 49
Sutton et al., 2019^21^	Abortion and/or EHM (n = 10)	RT qPCR	Overall (+): 5/10 horses	Overall (+): 2/10 horses	Total number of horses at facility: 179 of these 61 developed clinical signs. Data for horses that developed abortion and/or neurologic signs
Walter et al., 2013^22^	Respiratory signs: 17/64	Nested PCR	Overall (+): 0/64 horses	Overall (+): 0/64 horses	Data from Group 1 foals shown. Evaluated for the presence of other equine herpes viruses
Wang et al., 2007^23^	Respiratory signs: 17/64 None (N = 141)	Plaque assay EK cells	Overall (+): 0/64 horses	Overall (+): 8/64 horses	Data from Group 1 foals shown. Evaluated for the presence of other equine herpes viruses Data from Group 2 foals shown. Evaluated for the presence of other equine herpes viruses
		Multiplex Nested PCR	Overall (+): 0/141 horses	Overall (+): 6/141 horses	

*Note*: Bold italicized text indicates sample collected on the last day of sampling. Mean (±SD) data provided. Unless otherwise indicated, viremia was determined using PBMC samples.

Abbreviations: CAM, chorioallantoic membrane (chicken); cDNA, complementary DNA; EHM, equine herpesvirus‐1 myeloencephalopathy; gDNA, genomic DNA; ND, no data; NP, nasopharyngeal; PBMC, peripheral blood mononuclear cells; PCR, polymerase chain reaction (conventional PCR, analysis by electrophoresis); RT qPCR, real time quantitative PCR.

### 3.3 Description of the included experimental studies

A total of 60 experimental EHV‐1 infection studies met our inclusion criteria.^24^, ^25^, ^26^, ^27^, ^28^, ^29^, ^30^, ^31^, ^32^, ^33^, ^34^, ^35^, ^36^, ^37^, ^38^, ^39^, ^40^, ^41^, ^42^, ^43^, ^44^, ^45^, ^46^, ^47^, ^48^, ^49^, ^50^, ^51^, ^52^, ^53^, ^54^, ^55^, ^56^, ^57^, ^58^, ^59^, ^60^, ^61^, ^62^, ^63^, ^64^, ^65^, ^66^, ^67^, ^68^, ^69^, ^70^, ^71^, ^72^, ^73^, ^74^, ^75^, ^76^, ^77^, ^78^, ^79^, ^80^, ^81^, ^82^, ^83^ The key characteristics of these studies are reported in Table 2. Some studies evaluated vaccine or treatment efficacy in horses inoculated with EHV‐1. When treatments were performed only data from infected control animals was extracted and included in our analysis. Some studies performed experiments with 1 or more strains of virus or infection dose. A total of 47 studies used virus isolation methods to detect EHV‐1.^24^, ^26^, ^27^, ^28^, ^29^, ^30^, ^31^, ^32^, ^33^, ^34^, ^35^, ^38^, ^39^, ^40^, ^41^, ^42^, ^43^, ^44^, ^47^, ^48^, ^49^, ^50^, ^52^, ^54^, ^55^, ^56^, ^57^, ^58^, ^59^, ^60^, ^61^, ^62^, ^63^, ^64^, ^65^, ^66^, ^67^, ^68^, ^69^, ^70^, ^71^, ^74^, ^76^, ^77^, ^78^, ^79^, ^82^ In these studies, RK‐13 cells were used in the majority (66%) of the experiments. A total of 23 studies used PCR either as the only method or in combination with virus isolation.^25^, ^29^, ^36^, ^37^, ^44^, ^45^, ^46^, ^48^, ^51^, ^52^, ^53^, ^57^, ^60^, ^65^, ^66^, ^67^, ^72^, ^73^, ^75^, ^77^, ^78^, ^80^, ^81^, ^82^, ^83^ The great majority (22 of 24 used qPCR; Table 2).

**TABLE 2:** Main outcomes from experimental studies where both nasal samples and blood were evaluated.

Study	Breed	Total number of horses	Sex	Age	Viral challenge strain dose route	Virus isolation detection method	Clinical outcomes	Nasal shedding	Viremia
Bannai et al., 2018^24^	Thoroughbred	5 (controls)	F	16 to 21 mo	10‐I‐224 4 × 10^6^ pfu NR	Plaque assay RK‐13 cells	Fever: 5/5 Duration: 1.4 ± 0.4 d	Incidence: 5/5 Peak at 1 dpi (4/5), 2 dpi (1/5) Highest mean titer: 7.9 × 10^4^ pfu/mL	Incidence: 5/5 Duration = 3 ± 1 d Detected from 5 to 10 dpi Peak at 7 to 9 dpi
Bannai et al., 2023^25^	Thoroughbred	6 (controls)	F, M	15 to 18 mo	10‐I‐224 1 × 10^6^ pfu IN	RT PCR	Fever: 5/6 Duration: 1.7 ± 1.2 d	Incidence: 6/6 Peak at 1 dpi (4/6), 2 dpi (2/6) Highest mean: 6.6 Log_10_ copies/mL (1 dpi)	Incidence: 6/6 Duration = 7.3 ± 2.0 d Detected from 2 to 14 dpi Highest mean: 3.4 Log_10_ copies/mL (7 dpi)
Breathnach et al., 2001^26^	Mixed	2 (controls)	NR	5 to 7 mo	Army 183 2.35 × 10^7^ pfu IN	Plaque assay KyED cells	Fever: 2/2 Duration: 14 d	Incidence: 2/2 Duration: 9 d	Incidence: 2/2 Duration: 7.5 d
Breathnach et al., 2006^27^	Mixed	4	M	1 to 2 y	Army 183 7 × 10^7^ pfu IN	Plaque assay KyED cells	Fever: 3/4 Duration: 1.5 ± 1.3 d	Incidence: 4/4 Duration: 2.5 ± 0.6 d	Incidence: 1/4 Duration = 4 d (n = 1)
Bridges & Edington,1987^28^	Welsh Mtn	6	NR	Foals	Subtype 2 1 × 10^6.8^ TCID IN	Plaque assay EK cells	NR	Incidence: 6/6 Duration (1st inoculation): 8.2 ± 1.2 d	Incidence: 0/6 Duration (1st inoculation): 0 d
Brosnahan et al., 2010^29^	Mixed	4 (siLuc controls)	F (n = 2), CM (n = 2)	10 to 15 y	Ab4 1 × 10^7^ pfu IN	Plaque assay RK 13 cells	Fever: 4/4 Neurologic signs: 3/4	Incidence: 4/4 Peak median titer at 1 dpi: 7100 pfu/mL Detectable from 1 to 4 dpi	Incidence: 4/4 Detectable from 4 to 9 dpi
						RT qPCR		Incidence: 4/4 Peak median titer at 1 dpi: 8.0 × 10^8^ EHV‐1 genome copies/mL Median titer at 14 dpi: 6.4 × 10^4^ EHV‐1 genome copies/mL Detectable from 1 to 14 dpi	Incidence: 4/4 Peak median titer at 7 dpi: 2.4 × 10^4^ EHV‐1 genome copies/10^6^ β_2_M copies Median titer at 10 dpi: 166 EHV‐1 genome copies/10^6^ β_2_M copies Detectable from 1 to ***21*** dpi
Burki et al., 1990^30^	Haflinger Thoroughbred	2 (controls)	NR	NR	Piber 178/83 1 × 10^7^ TCID_50_ IN	Plaque assay RK‐13 cells	Fever: 2/2	Incidence: 2/2 Number positive: 1/2, 1/2, 0/2 @ 4, 6, 8 dpi	Incidence: 2/2 Number positive: 2/2, 1/2, 0/2, @ 4, 6, 8 dpi
Burrows et al., 1984^31^	Welsh Mtn	9 (controls)	PF (n = 9), NR (n = 11)	PF (5 to 17 y), 2‐year olds (n = 7), yearlings (n = 4)	3351/80 1 × 10^5.3^ pfu INH (aerosol)	Plaque assay RK‐13 cells	Fever: PF (8/9) Abortion (3/7)	Incidence PF: 5/9 Number positive: 5/9, 1/9, 1/9, 0/9, @ 4, 6, 8, 10 dpi Mean of positive samples: 1.32 × 10^3^ pfu	Incidence: 7/9 Number positive: 2/9, 6/9, 7/9, 1/9 @ 4, 6, 8, 10 dpi Mean of positive samples: 14.5 pfu/20 mL
		7 (controls)	NR	Two‐year olds			Fever: PF (7/7)	Incidence PF: 7/7 Number positive: 7/7, 7/7, 6/7, 3/7 @ 4, 6, 8, 10 dpi. Mean of positive samples: 7.08 × 10^5^ pfu	Incidence: 7/9 Number positive: 5/7, 7/7, 4/7, 3/7 @ 4, 6, 8, 10 dpi. Mean of positive samples: 25.7 pfu/20 mL
		4 (controls)	NR	Yearlings			Fever: PF (4/4)	Incidence PF: 4/4 Number positive: 4/4, 4/4, 4/4, 3/4 @ 4, 6, 8, 10 dpi. Mean of positive samples: 1.62 × 10^5^ pfu	Incidence: 7/9 Number positive: 3/4, 4/4, 3/4, 3/4 @ 4, 6, 8, 10 dpi. Mean of positive samples: 20.0 pfu/20 mL
Cornick et al., 1990^32^	NR	4 (controls)	NR	5 to 7 mo	Army 183 3 × 10^6^ pfu IN	Plaque assay Vero and BK cells	Fever: 4/4	Incidence: 4/4 Duration: 4.8 ± 2.6 d Detectable from 1 to 7 dpi	Incidence: 0/6 Duration: 0 d
Dolby et al., 1995^33^	Welsh cross	3 (controls)	NR	Yearlings	V592 1 × 10^6.9^ TCID_50_ IN	Plaque assay RK‐13 cells	Fever: 1/3	Incidence: 3/3 Duration: 1.3 ± 0.6 d Detectable from 2 to 13 dpi	Incidence: 3/3 Duration: 2 ± 1 d Detectable from 8 to 17 dpi
Eddington & Bridges 1990^34^	Welsh Mtn	6 (1st EHV‐1 challenge)	NR	Foals	Ab4 1 × 10^6.7^ TCID IN	Plaque assay EK cells	Fever: 6/6	Incidence: 6/6 Duration (1st inoculation): 6.0 ± 1.0 d	Incidence: 6/6 Duration (1st inoculation): 5.2 ± 0.8 d
Eddington et al., 1991^35^	Welsh Mtn	3	PF	7 to 10 y	Army 183 1 × 10^7.3^ TCID IN	Plaque assay EEK cells	Fever: 3/3. Paresis: 1/3 (@ 7 dpi) Aborted weak foals: 2/3) @ 12 to 14 dpi)	Incidence: 3/3 Duration: 4.7 ± 2.1 d	Incidence: 6/6 Duration: 4.3 ± 1.1 d
Foote et al., 2006^36^	Thoroughbred and Standardbred	4	PF	NR	HVS25A 4 × 10^7^ pfu INH (aerosol)	RT qPCR	Fever: 0/4	Incidence: 4/4 Duration: 5.3 ± 1.0 d Detectable from 2 to 10 dpi	Incidence: 4/4 Duration: 2.5 ± 1.3 d
			Foals				Fever: 4/4	Incidence: 4/4 Duration: 5.8 ± 2.9 d Detectable from 1 to 11 dpi	Incidence: 4/4 Duration: 2.5 ± 3.1 d
Gardiner et al., 2012^37^	NR	11	PF (n = 7) F (n = 4)	3 y	OH03 5 × 10^7^ pfu INH (aerosol)	RT qPCR	Fever: 11/11 Abortion: 1/7 (@ 12 dpi) Ataxia, hindlimb weakness: 1/11 (@ 5 to 6 dpi)	Incidence: 11/11 1 to 6 dpi, peak viral shedding 1 to 4 dpi Peak (PF): (1.25 ± 0.125) × 10^6^ gB copies/10^6^ β actin copies	Incidence: 11/11 5 to 8 dpi, peak viral shedding 5 to 8 dpi Peak (PF): (1.7 ± 1.2) × 10^3^ gB copies/10^6^ β actin copies
		9	PF (n = 7) F (n = 2)		Ab4 5 × 10^7^ pfu INH (aerosol)		Fever: 8/9 Abortion: 5/7 (@ 12 dpi)	Incidence: 9/9 1 to 9 dpi, peak viral shedding 2 dpi Peak (PF): (2.06 ± 4.57) × 10^5^ gB copies/10^6^ β actin copies	Incidence: 9/9 5 to 9 dpi, peak viral shedding 5 to 8 dpi Peak (PF): 801 ± 195 gB copies/10^6^ β actin copies
Garre et al., 2009^38^	Shetland pony	4 (controls)	M (n = 3), F (n = 1)	< 8 mo	03P37 1 × 10^6.5^ TCID_50_ IN/PO (50:50)	Plaque assay RK‐13 cells	Fever: 4/4	Incidence: 4/4 Detected from 1 to 7 dpi Peak: (6.3 ± 3.2) × 10^5^ TCID_50_/g nasopharyngeal mucus)	Incidence: 4/4 Detected from 2 to 14 dpi Peak number of infected cells able to transmit virus/10^7^ PBMC: 10 ± 5
Gibson et al., 1992^39^	Welsh Mtn	4 (primary infection)	M (n = 2), F (n = 2)	3 to 4 mo	Ab4 7.3 × 10^7^ pfu IN	Plaque assay RK‐13 cells	Fever: 4/4	Incidence: 4/4 Detected from 2 to 14 dpi Peak: 14500 ± 11 700 pfu/sample	Incidence: 4/4 Detected from 6 to 14 dpi Peak: 167 ± 147 IC/10^6^ cells
Gibson et al., 1992^40^	Welsh Mtn	1	F	3 mo	Ab4 2 × 10^7^ pfu IN	Plaque assay RK‐13 cells	Fever: 1/1	Incidence: 1/1 Detected from 2 to 8 dpi Peak: 8.7 × 10^4^ pfu/sample	Incidence: 0/1
		2	F, M		Ab4p 1 × 10^7^ pfu IN		Fever: 2/2	Incidence: 2/2 Detected from 2 to 8 dpi Peak: 9.2 × 10^4^ and 1.2 × 10^5^ pfu/sample	Incidence: 2/2 Detected from 3 to 15 dpi
Gibson et al., 1992^41^	Welsh Mtn	4 (primary infection)	NR	Foals	Ab4 7.3 × 10^7^ pfu IN	Plaque assay RK‐13 cells	Fever: 4/4	Incidence: 4/4 Detected from 1 to 14 dpi Peak (@ 1 and 8 dpi): 8.8 × 10^3^ pfu/sample	Incidence: 4/4 Detected from 6 to 9 dpi Peak: 9.2 × 10^4^ and 1.2 × 10^5^ pfu/sample
Gibson et al., 1992^42^	Welsh Mtn	3 (controls)	NR	3 to 4 mo	Ab4 1 × 10^7^ pfu IN	Plaque assay RK‐13 cells	Fever: 3/3	Incidence: 3/3 Detected from 1 to 12 dpi Peak: 91800 ± 11 800 pfu/sample	Incidence: 2/3 Detected from 3 to 11 dpi Peak: 10 to 1 IC/10^6^ cells
Gleeson & Coggins 1980^43^	Welsh Mtn Standardbred	11	PF	NR	Army 183 5 × 10^7.5^ TCID_50_ INH (aerosol)	Plaque assay FEK cells	Fever: 11/11 Abortion: 1/10	Incidence: 8/8 Detected from 2 to 9 dpi	Incidence: 10/11 Duration: 4.3 ± 2.5 d
		10			KyB 5 × 10^6.5^ TCID_50_ IN		Fever: 5/10 Abortion: 1/10 (@ 36 dpi)	Incidence: 5/10 Detected from 2 to 9 dpi	Incidence: 8/10 Duration: 3.5 ± 2.9 d
Goehring et al., 2010^44^	Standardbred	4	F	14 to 20 y	NR (neuropathogenic) 5 × 10^7.6^TCID_50_ IN	RT qPCR	Fever: 4/4 Weakness and ataxia: 2/4 (starting at 3 or 7 dpi)	Incidence: 4/4 Detected from 2 to ***14*** dpi Peak: (1.98 ± 2.61) × 10^7^ DNA copies/mL	Incidence: 1/4 Detected from 8 to 12 dpi Peak: 1.88 × 10^5^ DNA copies/2.5 × 10^6^ cells
						Plaque assay RK‐13 cells		Incidence: 0/4	Incidence: 1/4 Detected @ 9 dpi
Goehring et al., 2010^45^	Mixed	8 (controls)	M (n = NR), F (n = NR)	11 to 13 mo	OH03 5 × 10^7^ pfu INH (aerosol)	RT qPCR	Fever: 8/8	Incidence: 8/8 Detected from 1 to ***14*** dpi Peak (@ 2 dpi): 8.2 × 10^5^ gB copies	Incidence: 8/8 Detected from 5 to ***14*** dpi Peak (@ 7 dpi): 730 gB copies/10^6^ β actin copies
Goodman et al., 2007^46^	Welsh Mtn	4	F	2 y	Ab4 (D752) 1 × 10^7^ TCID_50_ INH (aerosol)	RT qPCR	Fever: NR Peak median (@ 2 dpi): 40.0°C Neurologic signs: 1/4	Incidence: NR Detected from 1 to ***14*** dpi Peak median (@ 4 dpi): 4.9 × 10^6^ gB copies	Incidence: NR Detected from 1 to 11 dpi Peak median (@ 6 dpi): 629 gB copies/10^6^ β actin copies
		4			Ab4 (N752) 1 × 10^7^ TCID_50_ INH (aerosol)		Fever: NR Peak median (@ 2 dpi): 38.8°C Neurologic signs: 0/4	Incidence: NR Detected from 1 to ***14*** dpi Peak median (@ 2 dpi): 2.6 × 10^6^ gB copies	Incidence: NR Detected from 4 to ***14*** dpi Peak median (@ 7 dpi): 41 gB copies/10^6^ β actin copies
	Mixed breed	7	CM (n = 2), F (n = 5)	3 to 16 y	Ab4 (D752) 1 × 10^7^ TCID_50_ INH (aerosol)		Fever: NR Peak median (@ 2 dpi): 39.9°C Neurologic signs: 2/7	Incidence: NR Detected from 1 to ***14*** dpi Peak median (@ 2 dpi): 8.0 × 107 gB copies	Incidence: NR Detected from 1 to ***14*** dpi Peak median (@ 6 dpi): 73 gB copies/10^6^ β actin copies
		7			Ab4 (N752) 1 × 10^7^ TCID_50_ INH (aerosol)		Fever: NR Peak median (@ 2 dpi): 39.6°C Neurologic signs: 0/4	Incidence: NR Detected from 1 to ***14*** dpi Peak median (@ 1 dpi): 6.3 × 10^7^ gB copies	Incidence: NR Detected from 2 to 10 dpi Peak median (@ 6 dpi): 14 gB copies/10^6^ β actin copies
Gryspeerdt et al., 2010^47^	Shetland pony	6	M	0.5 to 2 y	03P37 (NeurO) 1 × 10^6.5^ TCID_50_ IN/PO (50:50)	Plaque assay RK‐13 cells	Fever: 5/5	Incidence: 5/6 Detected from 1 to ***7*** dpi Peak (@ 2 dpi): 9.4 × 10^5^ TCID_50_/g	Incidence: 5/6 Detected from 1 to ***7*** dpi Peak (@ 6 dpi): 21 plaques/10^7^ cells (n = 2)
		6			97P70 1 × 10^6.5^ TCID_50_ IN/PO (50:50)		Fever: 5/5	Incidence: 6/6 Detected from 1 to ***7*** dpi Peak (@ 2 dpi): 4.5 × 10^5^ TCID_50_/g	Incidence: 6/6 Detected from 1 to ***7*** dpi Peak (@ 6 dpi): 37 plaques/10^7^ cells (n = 2)
Gupta et al., 2000^48^	NR	4	F	Adult	H7 15 × 10^6^ TCID_50_ IN/IV (2:1)	Plaque assay RK‐13 cells	Fever: 4/4	Incidence: 3/4 Detected from 1 to 7 dpi	Incidence: 0/4
						PCR		Incidence: 3/4 Detected from 1 to 7 dpi	Incidence: 0/4
Hannant et al., 1993^49^	Welsh Mtn	6 (controls)	NR	NR	V592 1 × 10^5^ TCID_50_ IN	Plaque assay RK‐13 cells	Fever: 6/6	Incidence: 6/6 Detected from 1 to ***10*** dpi Peak (@ 1 dpi): (8.8 ± 7.2) × 10^5^ TCID_50_/mL Duration: 7.8 ± 0.5 d	Incidence: 6/6 Detected from 2 to ***19*** dpi Peak (@ 8 dpi): 121.8 ± 251.1 TCID_50_/mL Duration: 11.5 d
Heldens et al., 2001^50^	Irish breeds	5 (controls)	Mixed	5 to 8 mo	121 412 1 × 10^5^ TCID_50_ IN	Plaque assay RK‐13 cells	Rectal temp: 39.4 ± 0.7°C	Incidence: 5/5 Duration: 10 ± 3.2 d Titer: 5.0 ± 8.3 TCID_50_/0.1 mL Peak (@ 3 dpi): 1.78 × 10^4^ TCID_50_/0.1 mL	Incidence: 4/5 Duration: 2.2 ± 1.5 d
	Welsh Mtn	4 (controls)	PF	3 y	Ab4 1 × 10^5^ TCID_50_ IN		Fever: 2/4 Ataxia: 1/4 (@ 9 dpi) Abortion: 4/4	Incidence: 5/5 Duration: 3.5 d Detected from 1 to 5 dpi Peak (@ 3 dpi): 3.16 × 10^5^ TCID_50_/mL	Incidence: 4/5 Duration: 4.3 d Detected from 1 to 21 dpi
Holz et al., 2017^51^	NR	8	Mixed	Yearling	Ab4 (WT) 5 × 10^7^ PFU IN	RT qPCR	Rectal temp: 40.2 ± 1.1°C Neurologic signs (@ 9 dpi): 3/8 Recumbent: 2/8	Incidence: 8/8 Peak (2 dpi): (3.19 ± 4.87) × 10^5^ gB copies	Peak (6 dpi): 706 ± 750 gB copies/10^5^ β actin copies
		9			Ab4 (N752) 5 × 10^7^ PFU IN		Rectal temp: 39.2 ± 0.9°C	Incidence: 9/9 Peak (1 dpi): (1.95 ± 3.09) × 10^5^ gB copies	Peak (7 dpi): 75 ± 87 gB copies/10^5^ β actin copies
		8			Ab4 (gD4) 5 × 10^7^ PFU IN		Rectal temp: 38.9 ± 0.6°C	Incidence: 8/8 Peak (4 dpi): (3.49 ± 9.88) × 10^4^ gB copies	Peak (10 dpi): 148 ± 144 gB copies/10^5^ β actin copies
Hussey et al., 2006^52^	NR	15	Mixed	1 to 2 y	Army 183 2 × 10^7^ PFU IN	RT qPCR	Fever: 15/15	Incidence: 15/15 Duration: 2 d Detected from 1 to 7 dpi Peak (@ 2 dpi): 2.1 × 10^3^ gB copies	Incidence: 0/15 Leucocyte samples
						Plaque assay ED cells		Incidence: 12/15 Duration: 4.9 d Detected from 1 to 4 dpi Peak (@ 2 dpi): 1420 ± 3430 PFU/mL (n = 6, one outlier) Peak @ 1 dpi: 250 ± 302 PFU/mL (n = 8)	Incidence: 9/15 Duration: 1 to 3 d Detected from 3 to 16 dpi
		3			Army 183 4 × 10^7^ PFU IN	RT qPCR	Fever: 3/3	ND	Incidence: 3/3 Duration: 4 to 14 d Detected from 4 to 14 dpi Peak @ 5 dpi: 180 ± 240 gB copies
						Plaque assay ED cells			PBMC samples Incidence: 1/3 Duration: 2 d Detected on 5 and 7 dpi
Hussey et al., 2013^53^	NR	6 (Experiment 3)	NR	9 to 18 mo	Ab4 5 × 10^7^ PFU INH (aerosol)	RT qPCR	Fever: 6/6 Median rectal temp: 41.0°C	Detected: 1 to 4 dpi Median peak @ 2 dpi: 2.48 × 10^6^ gB copies	Incidence: 6/7 Detected: 3 to 8 dpi Median peak @ 6 dpi: 1640 gB copies/10^6^ β actin copies
		6 (Experiment 3)			Ab4GFP 5 × 10^7^ PFU INH (aerosol)		Fever: 6/6 Median rectal temp: 40.8°C	Detected: 1 to 4 dpi Median peak @ 2 dpi: 4.24 × 10^6^ gB copies	Incidence: 7/7 Detected: 4 to 9 dpi Median peak @ 6 dpi: 4210 gB copies/10^6^ β actin copies
Kydd et al., 2003^54^	NR	9 (controls)	PF	3 to 15 y	Ab4 1 × 10^5^ PFU IN	Plaque assay RK‐13 cells	Fever: 9/9, duration: 1.8 ± 1.3 d Abortion: 9/9	Duration: 3.6 ± 1.1 d	Duration: 4.3 ± 1.9 d
Kydd et al., 2020^55^	Welsh Mtn	6 (controls)	F	5 to 7 mo	Ab4 2 × 10^6.3^ PFU INH (aerosol)	Plaque assay RK‐13 cells	Fever: 6/6	Incidence: 6/6 Duration: 8.3 ± 1.6 d Peak @ 2 dpi: (8.1 ± 73.0) × 10^5^ PFU/mL	Incidence: 6/6 Duration: 6.2 ± 1.0 d
Martens et al., 1989^56^	Quarter Horse	2	PF	NR	Army 183 3 × 10^6^ PFU IN	Plaque assay BK and ED cells	Fever: 2/2 Abortion: 1/2 (@ 15 dpi) Weak foal: 1/2 (@ 63 dpi)	Incidence: 2/2 Duration: 4 d Detected from 3 to 7 pdi	Incidence: 0/2
		2			Army 183 3 × 10^6^ PFU IN, IV (50:50)		Fever: 2/2	Incidence: 0/2	Incidence: 0/2
Maxwell et al., 2017^57^	Light horse breeds	6 (Controls)	F	> 20 y	Findlay OH 2003 (T953) 1 × 10^7^ PFU IN	Plaque assay BK cells	Fever: 6/6 Severe ataxia/euthanasia: 2/6 (@ 11 to 14 dpi)	Incidence: 6/6 Detected from 1 to 9 dpi Peak @ 1 dpi: (1.44 ± 2.16) × 10^5^ PFU/mL	ND
						RT qPCR		Incidence: 6/6 Detected: 1 to ***14*** dpi Peak @ 2 dpi: 1.23 × 10^9^ copies/mL	Incidence: 6/6 Detected: 1 to ***14*** dpi Peak @ 9 dpi: 2.28 × 10^4^ copies/10^6^ cells
Minke et al., 2006^58^	Welsh Mtn	5 (Controls)	Mixed	1 to 2 y	Ab4 1 × 10^5^ TCID_50_ IN	Plaque assay RK‐13 cells	Fever: 5/5	Incidence: 5/5 Duration: 5.4 d Peak @ 2 dpi: 7.08 × 10^5^ TCID_50_/mL	Incidence: 5/5 Duration: 19 d
Mohd‐Azmi et al., 2002^59^	Welsh Mtn	2 (Controls)	NR	NR	Ab4 1 × 10^7^ PFU IN	Plaque assay RK‐13 cells	Fever: 2/2	Incidence: 2/2 Detected: 1 to ***18*** dpi Peak @ 2 dpi: 4.03 × 10^5^ and 1.04 × 10^5^ PFU	Incidence: 2/2 Detected: 1 to 9 dpi Duration: 5 and 9 d
Mori et al., 2009^60^	NR	7	CM (n = 6), F (n = 1)	8 to 16 y	A4/72 1 × 10^6.6^ PFU IN	Plaque assay Vero cells	Fever: 0/7	Incidence: 0/7	Incidence: 0/7
						PCR		ND	Incidence: 7/7 Detected: 1 to ***30*** dpi
O'Neill et al., 1999^61^	NR	7	NR	1 to 2 y (n = 5) 7 y (n = 2)	Ab4 NR IN	Plaque assay ED cells	Fever: 3/7	Incidence: 5/7 Detected from 4 to 10 dpi Duration: 6.2 ± 2.7 d (n = 5)	Incidence: 7/7 Detected from 3 to 5 dpi Duration: 3.6 ± 0.8 d
Patel et al., 2003^62^	Hungarian half‐bred	8 (Controls)	Mixed	15 to 24 mo	C147 2 × 10^6^ TCID_50_ IN	Plaque assay ED cells	Fever: 8/8	Incidence: 8/8 Duration: 4.8 ± 1.5 d Titer: 400 ± 3 TCID_50_/sample	Incidence: 8/8 Duration: 2.9 d
Patel et al., 2003^63^	Welsh Mtn	6 (Controls)	PF	NR	C147 1 × 10^5.7^ TCID_50_ IN	Plaque assay ED cells	Fever: 6/6	Incidence: 6/6 Duration: 6.5 ± 0.8 d Titer: 3980 ± 3 TCID_50_/2 mL	Incidence: 6/6 Duration: 2.3 ± 0.5 d
Patel et al., 2004^64^	NR	6 (Controls)	NR	2 to 4 mo	C147 2 × 10^6^ TCID_50_ IN	Plaque assay ED cells	Fever: 6/6	Incidence: 6/6 Duration: 7.7 ± 2.9 d Titer: 630 TCID_50_/2 mL	Incidence: 6/6 Duration: 2.0 ± 1.5 d
Perkins et al., 2013^65^	NR	6 (siLuc Controls)	CM (n = 4), F (n = 2)	3 to 20 y	Ab4 1 × 10^7^ PFU IN	Plaque assay RK‐13 cells	Fever: 6/6 Ataxia: 2/6 (@ 6 to 9 dpi)	Incidence: 6/6 Detected: 1 to 5 dpi Peak: 2200 PFU/mL	Incidence: 6/6 Detected: 2 to 8 dpi
						RT qPCR		Incidence: 6/6 Detected: 1 to *21* dpi Median peak @ 1 dpi: 3.66 × 10^7^ copies/10^6^ cells (n = 6) Maximum: 3.9 × 10^7^ copies/10^6^ cells (n = 1)	Incidence: 6/6 Detected: 2 to ***21*** dpi Median peak @ 7 dpi: 7.50 × 10^3^ copies/10^6^ cells (n = 6) Maximum: 7735 copies/10^6^ cells (n = 1)
Perkins et al., 2019^66^	Icelandic	5 (Controls)	CM (n = 3), F (n = 2)	2.5 y	Ab4 1 × 10^7^ PFU INH (aerosol)	Plaque assay RK‐13 cells	Fever: 5/5	Incidence: 5/5 Detected: 1 to 5 dpi Peak @ 3 dpi: (3.09 ± 2.97) × 10^4^ PFU/mL	Incidence: 5/5 Detected: 1 to 10 dpi
						RT qPCR		ND	Incidence: 5/5 Detected: 4 to 9 dpi Peak @ 5 dpi: 31.9 ± 0.2 Ct
Schnabel et al., 2019^67^	Icelandic	8 (Controls)	CM (n = 4), F (n = 4)	3 to 4 y	Ab4 1 × 10^7^ PFU INH (aerosol)	Plaque assay RK‐13 cells	Fever: 8/8 Ataxia: 1/8 (@ 4 to 5 dpi)	Incidence: 8/8 Detected: 1 to 6 dpi Peak @ 2 dpi: (1.06 ± 1.98) × 10^5^ PFU/mL	Incidence: 8/8 Detected: 5 to 9 dpi Peak @ 6 dpi: 20.8 ± 16.7 PFU/10^7^ cells
						RT qPCR		ND	Incidence: 8/8 Detected: 4 to 8 dpi Peak @ 5 dpi: 35.0 ± 2.6 Ct
Seahorn et al., 1990^68^	Thoroughbred Quarter Horse	6 (Controls)	NR	5 to 7 mo	Army 183 3 × 10^5^ PFU IN	Plaque assay EK and BK cells	Fever: 5/6 Ataxia: 1/6 (@ 10 dpi)	Incidence: 6/6 Detected: 1 to 6 dpi Duration: 4.33 ± 0.94 d	Leucocytes Incidence: 3/6
Slater et al., 1993^69^	Welsh Mtn	2 (Wildtype)	NR	3 to 4 mo	Ab4 1 × 10^7^ PFU IN	Plaque assay RK‐13 cells	Fever: 2/2	Incidence: 2/2 Detected: 1 to 11 dpi Peak: 8.61 × 10^4^ PFU (1 dpi) or 1.45 × 10^4^ PFU (3 dpi)	Incidence: 2/2 Detected (≥1/10^5^ cells): several dpi
Smith et al., 2000^70^	Welsh Mtn	5	PF	10 to 18 y	V592 1 × 10^7.5^ TCID_50_ INH (aerosol)	Plaque assay RK‐13 cells	Fever: NR	Incidence: 5/5 Detected: 1 to 11 dpi Duration: 4.4 ± 0.5 d	Incidence: 3/5 Detected: 7 to 11 dpi Duration: 1.7 ± 0.6 d (n = 3)
Soboll et al., 2006^71^	NR	5 (Controls)	Mixed	1 y	Army 183 2 × 10^7^ PFU IN	Plaque assay ED cells	Fever: 5/5	Incidence: 4/5 Detected: 1 to 5 dpi Peak @ 1 dpi: 296 ± 331 PFU/mL	Incidence: 1/5 Detected: 16 dpi (n = 1) Duration: 1 d (n = 1)
Soboll et al., 2010^72^	NR	5 (Controls)	Mixed	2 to 3 y	Army 183 5 × 10^7^ PFU INH (aerosol)	RT qPCR	Mean clinical score > 2	Incidence: 5/5 Detected: 1 to 5 dpi Peak @ 1 dpi: 4.96 × 10^3^ gB copies	Incidence: 5/5 Detected: 6 to 13 dpi Peak @ 8 dpi: 194 gB copies/10^6^ β actin copies
Soboll Hussey et al., 2011^73^	NR	7	Mixed	Yearling	Ab4 (WT) 1 × 10^7^ PFU IN	RT qPCR	Fever: 7/7	Incidence: 5/5 Detected: 1 to 8 dpi Peak @ 2 dpi: 8530 ± 9990 gB copies	Incidence: 5/5 Detected: 6 to 9 dpi Peak @ 7 dpi: 328 ± 864 gB copies/10^6^ β actin copies
					Ab4 ΔORF1/2 1 × 10^7^ PFU IN		Fever: 7/7	Incidence: 5/5 Detected: 1 to 4 dpi Peak @ 2 dpi: 342 ± 1770 gB copies	Incidence: 5/5 Detected: 6 to 9 dpi Peak @ 7 dpi: 120 ± 492 gB copies/10^6^ β actin copies
Stokes et al., 1991^74^	NR	3	NR	18 mo	3551/80 1 × 10^6^ TCID_50_ IN	Plaque assay RK‐13 cells	Fever: 3/3	Incidence: 3/3 Detected: 1 to 19 dpi Peak @ 2 dpi: (1.47 ± 1.66) × 10^7^ TCID_50_/mL	ND
						Plaque assay EK cells		Incidence: 3/3 Detected: 1 to 11 dpi Peak @ 2 dpi: (2.43 ± 0.5) × 10^5^ TCID_50_/mL	Incidence: 3/3 Detected: 3 to 11 dpi
Sutton et al., 2020^75^	Welsh Mtn	4	NR	10 mo	FR‐56628 NR INH	RT qPCR	Fever: 4/4 Tail hypotonia: 4/4 (@ 2 to 7 dpi)	Incidence: 3/3 Detected: 1 to ***20*** dpi Peak @3 dpi: (1.63 ± 4.32) × 10^7^ copies/mL	Incidence: 3/3 Detected: 3 to ***20*** dpi Peak @9 dpi: (1.61 ± 2.95) × 10^5^ copies/mL
Tewari et al., 1993^76^	Welsh Mtn	1 (Primary infection)	NR	3 to 4 mo	Ab4 1 to 5 × 10^7^ PFU IN	Plaque assay RK‐13 cells	Fever: 1/1	Incidence: 1/1 Duration: 11 d (n = 1)	Incidence: 1/1
Thieulent et al., 2022^77^	Welsh Mtn	4 (Controls)	M	8 mo	FR‐56628 5 × 10^7^ TCID_50_ INH	Plaque assay RK‐13 cells	Fever: 4/4	Incidence: 4/4 Detected: 1 to 12 dpi Peak @6 dpi mean = 4.05 ± 0.50 TCID_50_/mL	Incidence: 4/4 Detected: 4 to 11 dpi Peak @7 dpi mean = 9.7 Log_10_ copies/mL (cell lysate)
						RT qPCR		Incidence: 4/4 Detected: 1 to 20 dpi Peak @4 dpi mean = 7.2 × 10^7^ copies/mL	Incidence: 4/4 Detected: 3 to 20 dpi Peak @9 dpi = 4.14 ± 0.42 Log_10_ copies/2 × 10^6^ PBMC
Tsujimura et al., 2009^78^	Thoroughbred	2	NR	51 to 59 d	89C25p (ΔgE) 1 × 10^7^ PFU IN	Plaque assay EK or BK cells	Fever: 0/2	Incidence: 0/2	Incidence: 0/2
						RT qPCR		Incidence: 2/2 Duration: 6 d Detected: 1 to 6 dpi Peak @ 1 to 2 dpi: 630 to 5000 gene copies	Incidence: 0/2
		2		52 to 59 d	89C25p (gE rev) 1 × 10^6^ PFU IN	Plaque assay EK or BK cells	Fever: 2/2	Incidence: 2/2 Duration: 7 to 8 d Detected: 1 to 8 dpi	Incidence: 2/2 Detected: 2 to ***14*** dpi
						RT qPCR		Incidence: 2/2 Duration: ***11*** d Detected: 1 to ***14*** dpi Peak @ 3 or 5 dpi: 1.5 × 10^6^ or 5.0 × 10^6^ gene copies	Incidence: 2/2 Duration: ***11*** d Detected: 1 to ***14*** dpi Peak @ 10 or 12 dpi: 630 or 2500 gene copies
		2		56 to 79 d	89C25p (ΔgE) 4 × 10^5^ PFU IM	Plaque assay EK or BK cells	Fever: 0/2	Incidence: 0/2	Incidence: 0/2
						RT qPCR		Incidence: 0/2	Incidence: 0/2
		3 (Controls)		25 to 80 d	89C25p 1 × 10^6^ PFU IN	RT qPCR	Fever 3/3	Incidence: 3/3 Detected: 1 to ***14*** dpi Peak @ 4 dpi: (8.2 ± 16.1) × 10^6^ gene copies/template DNA	Incidence: 3/3 Detected: 1 to ***14*** dpi Peak @ 4 dpi: 1030 ± 670 gene copies//10^6^ β_2_‐M copies
Van der Meulen et al., 2006^79^	Shetland pony	6	NR	9 mo to 20 y	97P70 1 × 10^6.5^ TCID_50_ IN	Plaque assay RK 13 cells	Fever: 4/6	Incidence: 6/6 Detected: 1 to 14 dpi Peak @ 1 dpi: (2.14 ± 3.22) × 10^5^ TCID_50_/g	Incidence: 6/6 Detected: 5 to 21 dpi Peak @ 5 dpi: 10.2 ± 7.2 infected cells/1 × 10^7^ PBMC (n = 5)
Van de Walle et al., 2010^80^	NR	4 (Controls)	NR	12 to 18 y	OH03 1 × 10^7^ PFU IN	RT qPCR	Fever: 4/4	Incidence: 4/4 Detected: 1 to ***14*** dpi Peak @ 3 dpi: (2.3 ± 5.1) × 10^8^ gene copies	Incidence: 4/4 Detected: 3 to 13 dpi Peak @ 7 dpi: 6160 ± 5840 gene copies//10^7^ β_2_‐M copies
Wagner et al., 2017^81^	Icelandic	5 (Controls)	M (n = 4), F (n = 1)	7 mo	NY03 1 × 10^7^ PFU INH (aerosol)	RT qPCR	Fever: 5/5	Incidence: 5/5 Detected: 1 to ***14*** dpi Peak @ 2 dpi: (5.0 ± 5.4) × 10^7^ gB gene copies/mL	Incidence: 5/5 Detected: 3 to ***1*** 4 dpi Peak @ 6 dpi: 31.8 ± 1.1 gB Ct value
Wimer et al., 2018^82^	Icelandic	5	CM (n = 3), F (n = 2)	2.5 y	Ab4 1 × 10^7^ PFU INH (aerosol)	Plaque assay RK 13 cells	Fever: 5/5	Incidence: 5/5 Detected: 1 to 4 dpi Duration: 2.4 ± 1.1 d Peak @ 1 dpi: 1460 ± 2540 PFU	Incidence: 5/5 Detected: 4 to 8 dpi Peak @ 6 dpi: 24.5 ± 35.6 PFU/10^7^ PBMC
						RT qPCR		ND	Incidence: 5/5 Detected: 4 to 9 dpi Peak @ 5 dpi: 33.4 ± 0.9 PFU/10^7^ PBMC
		5	CM (n = 4), F (n = 1)		Ab4ΔORF1/71 1 × 10^7^ PFU INH (aerosol)	Plaque assay RK 13 cells	Fever: 5/5	Incidence: 1/5 Detected: 1 to 2 dpi (n = 1) Peak @ 1 dpi: 200 PFU (n = 1)	Incidence: 5/5 Detected: 4 to 8 dpi Peak @ 6 dpi: 10.7 ± 16.3 PFU/10^7^ PBMC
						RT qPCR		ND	Incidence: 5/5 Detected: 4 to 7 dpi Peak @ 6 dpi: 32.8 ± 1.1 Ct/5 × 10^6^ PBMC
Zarski et al., 2021^83^	NR	7	M (n = 5), F (n = 2)	1 y	Ab4 5 × 10^7^ PFU IN	RT qPCR	Fever: 7/7 Neurologic signs: 1/7	Incidence: 5/5 Detected: 1 to 6 dpi Peak @ 2 dpi: 1.82 × 10^6^ copies/100 ng DNA (n = 1)	Incidence: 5/5 Detected: 3 to ***10*** dpi Peak @ 5 dpi: 950 copies/500 ng DNA (n = 1)

*Note*: Bold italicized text indicates sample collected on the last day of sampling. Unless otherwise noted, mean (±SD) data provided. Unless otherwise noted, viremia was performed using PBMCs.

Abbreviations: BK, bovine kidney; Ct, cycle threshold; ED, equine dermal; EEK, equine embryonic kidney; EK, equine kidney; FEK, fetal equine kidney; IC, infectious center; IN, intranasal; INH, inhalation; ND, not determined; PBMC, peripheral blood mononuclear cell; PCR, polymerase chain reaction (conventional PCR, analysis by electrophoresis); RK, rabbit kidney; RT qPCR, real time quantitative PCR.

### 3.4 Nasal shedding

**FIGURE 2:**
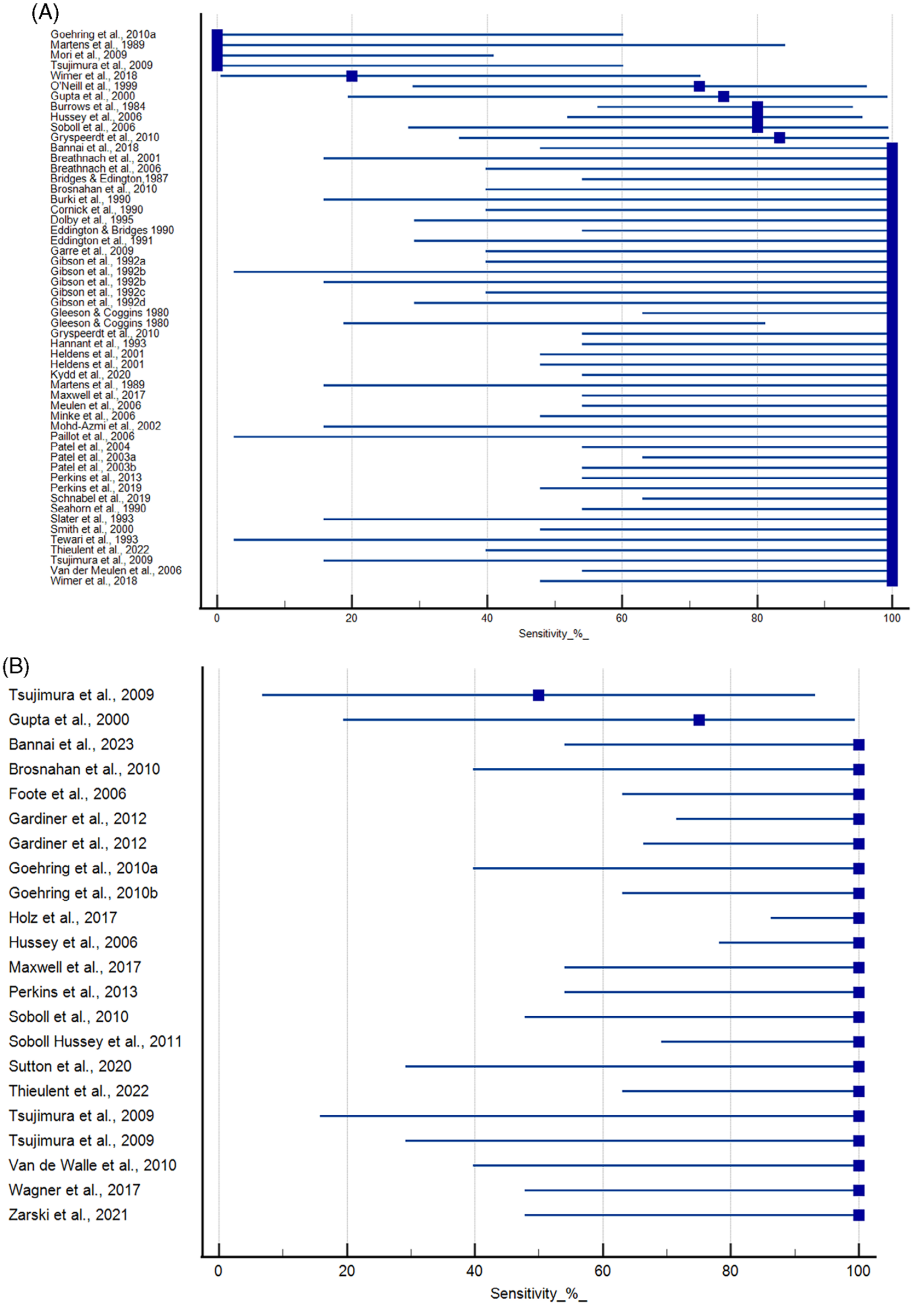
Sensitivity of virus isolation (A) and qPCR (B) for the detection of EHV‐1 in nasal swab samples from horses experimentally inoculated with EHV‐1. Duplicate entries for the same study represent results using different strains of virus or exposures. Mean and 95% confidence intervals provided.

Experimental studies that evaluated nasal shedding using virus isolation in cultured cells are shown in Figure 2A. Four studies failed to detect virus in nasal swab samples using virus isolation.^44^, ^56^, ^60^, ^78^ In 1 case, PCR detected EHV‐1 DNA in the nasal swab samples.^44^ Combined intranasal and intravenous administration of EHV‐1 strain Army 183 to horses resulted in negative nasal and PBMC samples.^56^ In contrast, administration of the virus solely by intranasal inoculation yielded positive results for the nasal swab samples in this study.^56^ Mori and coauthors used the A4/72 strain and Vero cells in their study and failed to detect virus in either nasal swab or PBMC samples.^60^ Only 1 other study used Vero cells. In this study, virus was successfully isolated from both nasal swab and PBMC samples.^32^ Wimer and coauthors report a low incidence of nasal shedding after infection with the Ab4ΔORF1/71 strain, while all animals exposed to the wildtype virus (Ab4) displayed nasal shedding.^82^ Sensitivity in the remaining studies was ≥70%, with most studies (60%) having 100% sensitivity. There were 19 studies that evaluated nasal shedding using PCR (Figure 2B).^25^, ^29^, ^36^, ^37^, ^44^, ^45^, ^48^, ^51^, ^52^, ^57^, ^65^, ^72^, ^73^, ^77^, ^78^, ^80^, ^81^, ^82^, ^83^ The majority (17/19) of studies reported 100% sensitivity using this method.

### 3.5 Viremia

**FIGURE 3:**
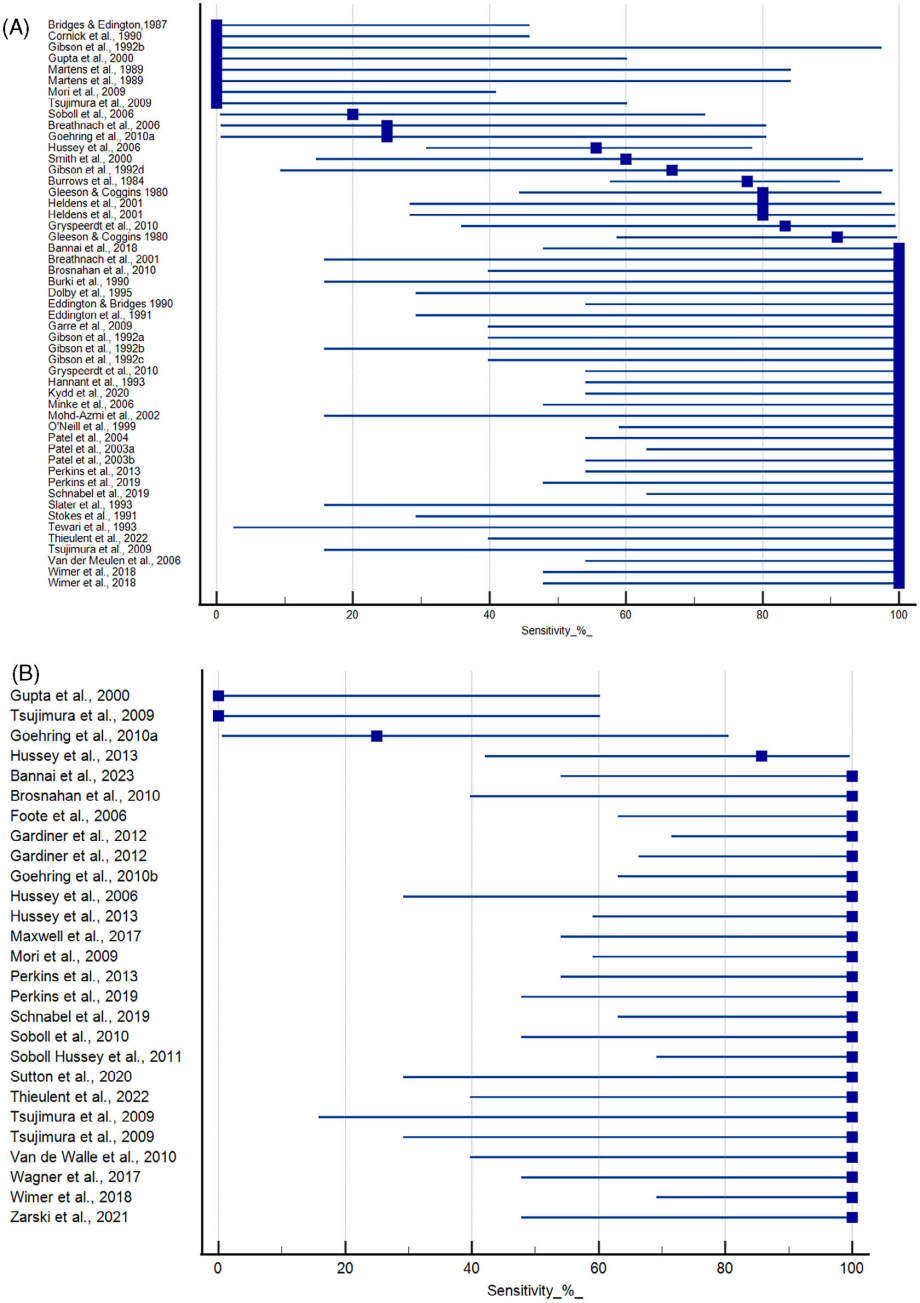
Sensitivity of virus isolation (A) and qPCR (B) for the detection of EHV‐1 in PBMC samples from horses experimentally inoculated with EHV‐1. Duplicate entries for the same study represent results using different strains of virus or exposures. Mean and 95% confidence intervals provided.

Experimental studies that evaluated viremia using blood samples and/or virus isolation are shown in Figure 3A. Seven experimental studies failed to detect virus in blood samples.^28^, ^32^, ^40^, ^48^, ^56^, ^60^, ^78^ Sensitivity in the remaining studies varied from 20% to 100%. The majority of the studies (72%) had 100% sensitivity using viral isolation methods on either PBMC or whole blood samples.^25^, ^26^, ^29^, ^30^, ^33^, ^34^, ^35^, ^38^, ^39^, ^40^, ^41^, ^47^, ^49^, ^55^, ^58^, ^59^, ^62^, ^63^, ^64^, ^65^, ^66^, ^67^, ^69^, ^74^, ^76^, ^77^, ^78^, ^79^, ^82^ Experimental studies that evaluated viremia using qPCR are shown in Figure 3B. The majority (19/23) of these studies demonstrated 100% sensitivity using this method.^25^, ^29^, ^36^, ^37^, ^45^, ^52^, ^53^, ^57^, ^60^, ^65^, ^66^, ^67^, ^72^, ^73^, ^75^, ^77^, ^79^, ^80^, ^82^

### 3.6 qPCR vs virus isolation to detect EHV‐1

**FIGURE 4:**
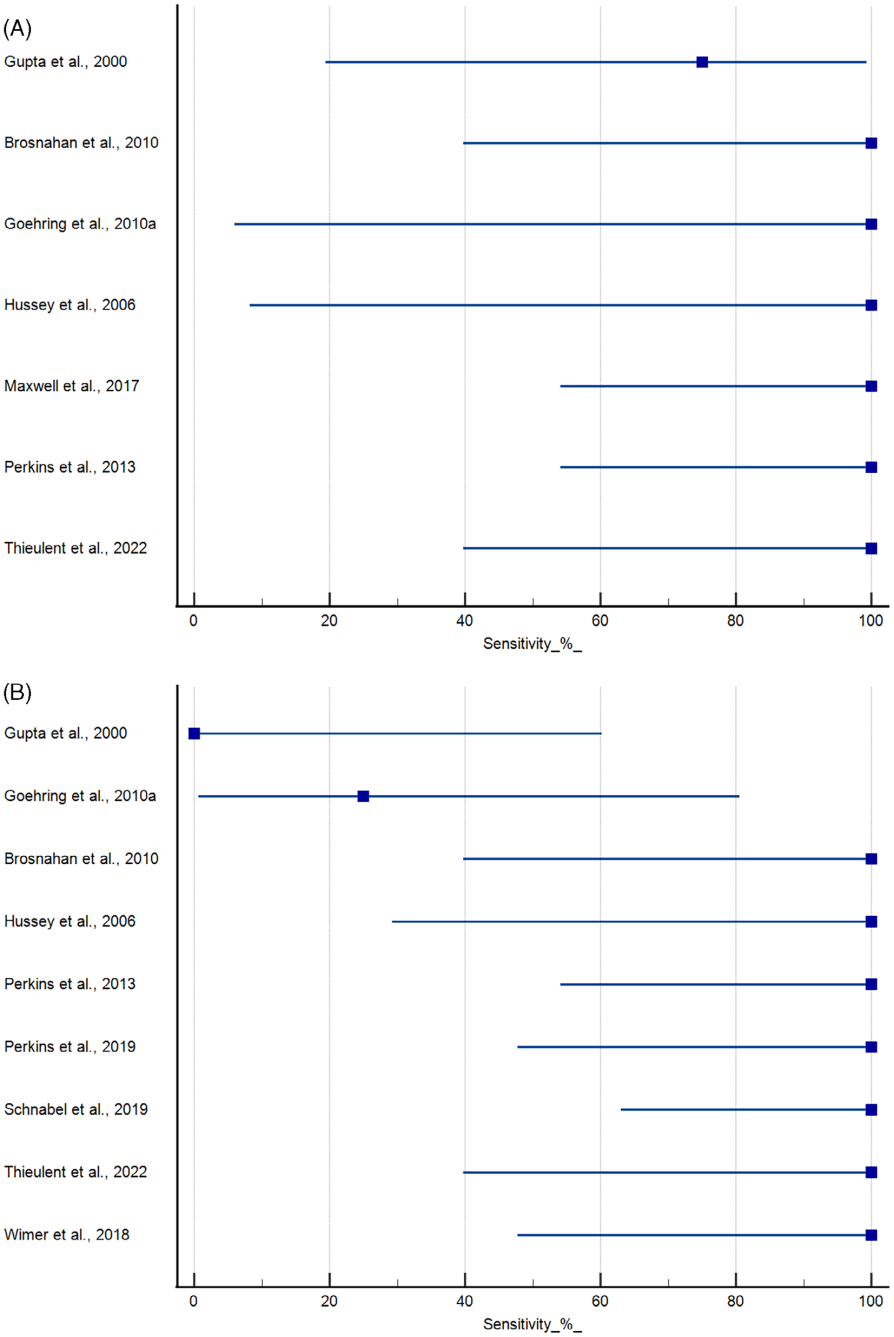
Relative sensitivity of qPCR vs virus isolation for the detection of EHV‐1 in either nasal swab (A) or PBMC samples (B) collected from horses experimentally inoculated with EHV‐1. Mean and 95% confidence intervals provided.

There were 6 experimental studies that measured nasal shedding using both virus isolation and qPCR (Figure 4A).^29^, ^44^, ^48^, ^52^, ^57^, ^65^ The sensitivity of qPCR matched or exceeded that of virus isolation techniques. Goehring and coauthors failed to detect EHV‐1 in nasal swab samples using virus isolation methods, whereas qPCR identified nasal shedding in all horses (n = 4) exposed to a neuropathogenic EHV‐1 strain.^44^ Virus isolation and qPCR had 80% and 100% sensitivity, respectively, in a study that exposed horses to the Army 183 strain.^52^ Both virus isolation and qPCR had a 75% sensitivity for nasal shedding in another study using 4 horses and an H7 strain of EHV‐1.^48^

There were 9 studies that evaluated viremia after purification of PBMC using both virus isolation and qPCR (Figure 4B).^29^, ^44^, ^48^, ^52^, ^65^, ^66^, ^67^, ^77^, ^82^ The sensitivity of qPCR matched or exceeded that of virus isolation in most cases. Virus isolation and qPCR failed to detect virus PBMC samples from 4 horses exposed to the H7 strain of EHV‐1.^48^ Both qPCR and virus isolation had 25% sensitivity in detecting EHV‐1 in PBMC samples in 4 horses exposed to a neuropathogenic strain of EHV‐1.^44^ Virus isolation and qPCR had 33% and 100% sensitivity, respectively, at detecting viremia in horses exposed to the Army 183 strain.^52^ In a second arm of this study, qPCR was unable to detect EHV‐1 from isolated leukocytes exposed to the Army 183 strain. Hussey and coauthors reported that the yield and quality of DNA prepared from the leukocyte were poor based on spectrophotometric analysis and agarose gels.^52^ Because of this technical concern, these data are not included in Figure 4B. In all other cases, both virus isolation and qPCR had a 100% sensitivity for the detection of EHV‐1 in PBMC samples.

### 3.7 Relative sensitivity of EHV‐1 detection in nasal swabs or blood samples following experimental inoculation

**FIGURE 5:**
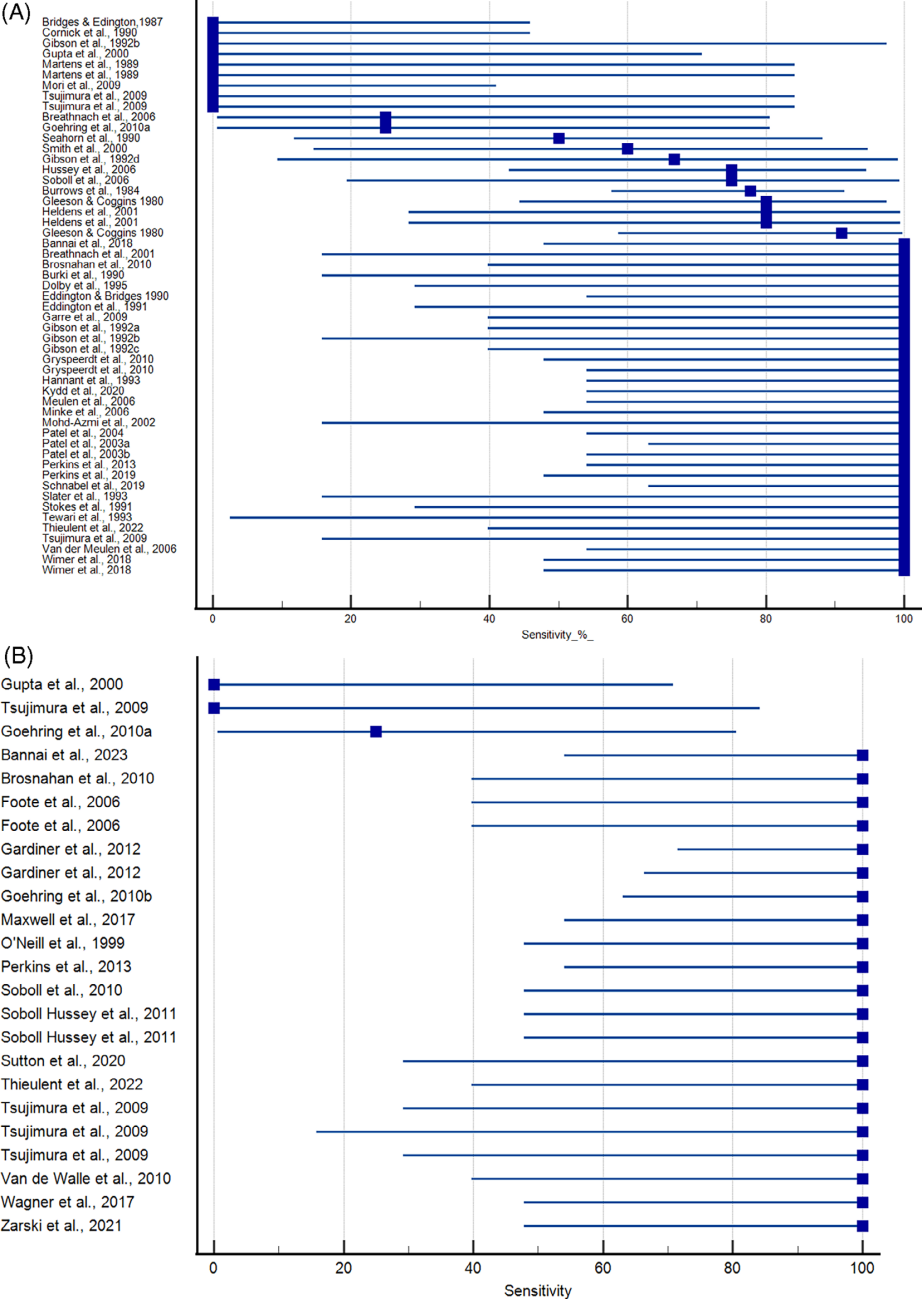
Relative sensitivity of nasal swabs or PBMC samples for the detection of EHV‐1 DNA in horses experimentally inoculated with EHV‐1. Data using cell culture‐based virus isolation methods (A) or (q)PCR (B) are shown. Sensitivity <100% indicates that detection rates in nasal swabs were higher than that seen in PBMC samples. Mean and 95% confidence intervals provided.

Experimental studies that used virus isolation methods to detect the presence of EHV‐1 in both nasal swab and blood samples are shown in Figure 5A. Seven studies using cell culture‐based virus isolation methods reported 100% sensitivity for the detection of EHV‐1 in nasal swabs, while failing to detect the virus in the PBMC samples.^28^, ^32^, ^40^, ^48^, ^56^, ^60^, ^78^ Nine other studies presented intermediate sensitivities (25%‐91%) for the detection of EHV‐1 in both nasal swabs and PBMC samples using virus isolation methods.^31^, ^42^, ^43^, ^44^, ^50^, ^52^, ^68^, ^70^, ^71^ The remaining studies had 100% sensitivity for the detection of EHV‐1 in both nasal swabs and PBMC samples using cell culture‐based virus isolation methods.^25^, ^26^, ^29^, ^30^, ^33^, ^34^, ^35^, ^38^, ^39^, ^40^, ^41^, ^47^, ^49^, ^55^, ^58^, ^59^, ^61^, ^62^, ^63^, ^64^, ^65^, ^66^, ^67^, ^69^, ^74^, ^79^, ^82^

Experimental studies that used qPCR to detect the presence of EHV‐1 DNA in both nasal swab and PBMC samples are shown in Figure 5B. Two studies reported 100% sensitivity for PCR‐based detection of EHV‐1 DNA in nasal swabs, while failing to detect the viral DNA in the blood (PBMC) samples.^48^, ^78^ Goehring and coauthors used qPCR to detect EHV‐1 DNA in nasal swab and blood (PBMC) samples from horses inoculated with a neuropathogenic strain of EHV‐1.^44^ In this study, the assay sensitivities were 100% and 25% for nasal swab and PBMC samples, respectively.^44^ All other studies had 100% sensitivity for the detection of EHV‐1 DNA in both nasal swabs and PBMC samples using qPCR.^25^, ^29^, ^36^, ^37^, ^45^, ^57^, ^61^, ^65^, ^72^, ^73^, ^75^, ^77^, ^80^, ^81^, ^82^, ^83^

### 3.8 Duration and magnitude of nasal shedding and viremia in experimental studies

**FIGURE 6:**
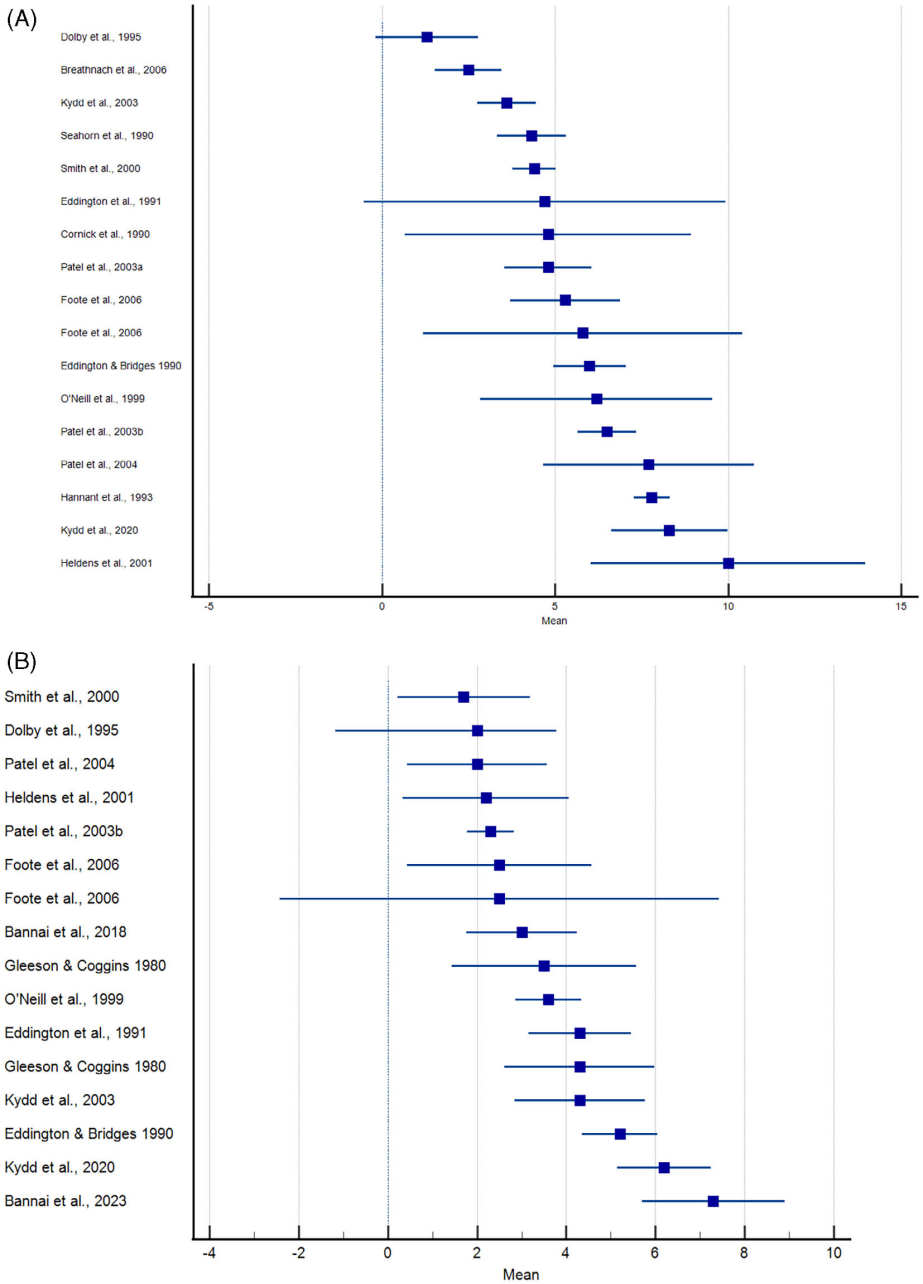
Mean duration ±95% confidence interval (days) of nasal shedding (A) and viremia (B) in horses experimentally inoculated with EHV‐1.

Figure 6 provides mean values and 95% confidence intervals for duration of nasal shedding and viremia. Data from studies using either cell culture‐based virus isolation methods or qPCR are included. Detection of nasal shedding generally occurred within 1 to 2 days after inoculation of horses with EHV‐1 (Table 1). Mean days of nasal shedding ranged from 1.3 to 10 days, with most studies reporting a duration of 3 to 7 days.^27^, ^32^, ^33^, ^34^, ^35^, ^36^, ^49^, ^50^, ^54^, ^55^, ^61^, ^62^, ^63^, ^64^, ^68^, ^70^ Nasal shedding decreased over time and remained detectable in some horses for more than 14 days after inoculation.^29^, ^41^, ^44^, ^45^, ^46^, ^57^, ^59^, ^65^, ^74^, ^75^, ^78^, ^79^, ^80^, ^81^ Viremia was often initially detected 1 or more days after nasal shedding was first seen. The duration of viremia was generally shorter in most studies, with mean durations of 2 to 4 days (range of means: 1.7 to 7.3 days).^24^, ^25^, ^32^, ^33^, ^34^, ^35^, ^36^, ^43^, ^50^, ^54^, ^55^, ^61^, ^63^, ^64^, ^70^ Viremia also decreased over time and remained detectable in some horses ≥21 days after inoculation (Table 1).^29^, ^50^, ^60^, ^65^, ^75^, ^79^

In the majority of experimental studies, duration and/or range of EHV‐1 detection in nasal secretions by culture and/or PCR was reported.^24^, ^25^, ^26^, ^27^, ^28^, ^29^, ^32^, ^33^, ^34^, ^35^, ^36^, ^37^, ^38^, ^39^, ^40^, ^41^, ^42^, ^43^, ^44^, ^45^, ^46^, ^47^, ^48^, ^49^, ^50^, ^52^, ^53^, ^54^, ^55^, ^56^, ^57^, ^58^, ^59^, ^61^, ^62^, ^63^, ^64^, ^65^, ^66^, ^67^, ^68^, ^69^, ^70^, ^71^, ^72^, ^73^, ^74^, ^75^, ^76^, ^77^, ^78^, ^79^, ^80^, ^81^, ^82^, ^83^ The detection of EHV‐1 in nasal secretions via culture was successful between 1 and 19 days after infection, with the mean duration ranging from 1.3 to 11 days. Detection of EHV‐1 in nasal secretions by qPCR was possible between 1 and 21 days, with the mean duration ranging from 2 to 11 days.

In a total of 52 studies, the duration and/or range of EHV‐1 detection in blood by culture and/or qPCR was reported.^24^, ^25^, ^26^, ^27^, ^28^, ^29^, ^32^, ^33^, ^34^, ^35^, ^36^, ^37^, ^38^, ^39^, ^40^, ^41^, ^42^, ^43^, ^44^, ^45^, ^46^, ^47^, ^49^, ^50^, ^52^, ^53^, ^54^, ^55^, ^57^, ^58^, ^59^, ^60^, ^61^, ^62^, ^63^, ^64^, ^65^, ^66^, ^67^, ^70^, ^71^, ^72^, ^73^, ^74^, ^75^, ^77^, ^78^, ^79^, ^80^, ^81^, ^82^, ^83^ The detection range of EHV‐1 in blood via virus isolation was between 1 and 21 days after infection, with a mean duration of 1 to 11.5 days. EHV‐1 was detected in blood via qPCR between 1 and 30 days after infection, with a mean duration ranging from 2 to 11 days.

Seven studies directly compared cell culture isolation and qPCR techniques for the detection of EHV‐1 in nasal secretions.^29^, ^48^, ^52^, ^57^, ^65^, ^77^, ^78^ The duration of EHV‐1 shedding in nasal secretions ranged from 0 to 9 days as assessed by cell culture isolation and from 0 to 21 days by qPCR. Eight studies had direct comparison of EHV‐1 detection in blood between virus isolation in cell culture and qPCR.^29^, ^52^, ^65^, ^66^, ^77^, ^78^, ^82^ The duration of EHV‐1 viremia ranged from 0 to 14 days as determined by virus isolation and from 0 to 30 days by qPCR detection of the virus.

## 4 DISCUSSION

Equine practitioners often face diagnostic challenges when presented with an equid that is potentially infected with EHV‐1. The disease stage following EHV‐1 infection influence viral kinetics in both blood and nasal secretions. Veterinarians routinely use nasal secretions (swabs) for the detection of EHV‐1 in horses with either fever or respiratory signs as well as acute onset of neurological disease (ie, suspect EHM case). Blood is less frequently collected for EHV‐1 detection but is used to corroborate presence of EHV‐1 during EHM outbreaks as viremia is considered a prerequisite for the development of neurological disease.^84^ The primary goal of the present study was to determine whether nasal secretions were a better‐suited biological sample when compared to blood for the success of detection of EHV‐1 infection. This should allow us to make science‐based recommendations to maximize the detection frequency of EHV‐1 in infected horses.

Most observational studies focused on horses with fever and respiratory signs or on horses with either neurological signs consistent with EHM or epidemic abortions. Our review identified a broad range of detection frequencies of EHV‐1 in sick equids following natural infection, likely reflecting the disease stage at which testing occurred, and the disease setting (ie, respiratory infection, abortion, EHM). The review clearly demonstrated that nasal secretions were consistently more successful in the detection of EHV‐1 in equids with fever and respiratory signs as well as in horses with suspected EHM. In contrast, when testing mares postabortion, EHV‐1 was more frequently detected in blood samples when compared to nasal secretions. These findings are consistent with the biology and pathogenesis of EHV‐1 as it consecutively first invades the respiratory apparatus, then the central nervous system, and finally the pregnant uterus. The incubation time for abortions is the longest for all clinical presentations of EHV‐1 infection.^1^

Collectively, observational and experimental studies support the use of nasal secretions as a biological sample that offers the highest chance of EHV‐1 detection when compared to blood. However, detection of EHV‐1 infection using either nasal swab or PBMC samples also depends upon the timing of sample collection. Viremia was often initially detected 1 or more days after nasal shedding was first seen. Thus, early detection of EHV‐1 in nasal secretions may be more sensitive overall. Within several days, both nasal shedding and viremia can occur concurrently, and detection may persist for several days to weeks thereafter. Repeated sampling of horses in potential outbreaks may increase the detection rate of EHV‐1 infections.

A secondary goal of this study was to evaluate how long virus could be detected after primary infection by qPCR. The detection of EHV‐1 by qPCR has become the diagnostic platform of choice for the past 2 decades and has supplanted virus isolation. Nucleic‐acid based detection systems have many diagnostic advantages such as high sensitivity and specificity, quick turn‐around‐time, and cost‐effectiveness. However, qPCR has several technical limitations, including risk of false‐positive and ‐negative results, and inability to differentiate between viable and nonviable virus.^3^ Technical advances in molecular diagnostics have overcome some of these limitations by using closed‐tubes assays and incorporating quality controls.^85^ In experimental studies with both nasal secretions and blood available for qPCR testing, detection of EHV‐1 in nasal secretions and blood ranged from 1 to 21 and 1 to 30 days postinfection, respectively, with similar duration of detection of approximately 9 days in either case. Virus was sometimes identified significantly later after initial infection and particularly in naturally occurring outbreaks, and the biological significance of these results are uncertain. Quantification of mRNA transcripts, a result of productive virus replication, could be used to determine the clinical significance of this phenomenon.^15^ Interpretation of qPCR test results should be done in the context of clinical signs of infected horses and unexpected or equivocal results should be investigated and repeated.

An important limitation here is our use of a narrative review approach to evaluate the literature, which incorporated many elements of a systematic review process. The main difference between a narrative and a systematic review relates to the absence of an evaluation of the study quality of individual studies.

In conclusion, the results of this study indicate that, under experimental conditions when animals are sampled repeatedly, both blood and nasal secretions display similar sensitivity with respect to the detection of EHV‐1. However, observational studies suggest that nasal secretions were consistently superior for detection of EHV‐1 in equids with fever and respiratory signs and in horses with suspected EHM. Experimental studies have also shown that EHV‐1 can be detected in both blood and nasal secretions by qPCR in most cases for approximately 9 days, with viremia being initially detected 1 or more days after nasal shedding is first detected. While it is very important to utilize highly sensitive diagnostic modalities such as qPCR, clinicians should interpret the diagnostic results in the clinical context.

## CONFLICT OF INTEREST DECLARATION

Authors declare no conflict of interest.

## OFF‐LABEL ANTIMICROBIAL DECLARATION

Authors declare no off‐label use of antimicrobials.

## INSTITUTIONAL ANIMAL CARE AND USE COMMITTEE (IACUC) OR OTHER APPROVAL DECLARATION

Authors declare no IACUC or other approval was needed.

## HUMAN ETHICS APPROVAL DECLARATION

Authors declare human ethics approval was not needed for this study.

## Supporting information


**Data S1.** Supporting Information.

**Data S2.** Supporting Information.
