# Supplementary material for: Viremia and nasal shedding for the diagnosis of equine herpesvirus‐1 infection in domesticated horses
Source: J Vet Intern Med. 2023 Dec 9;38(3):1765–91. doi: 10.1111/jvim.16958 (PMC11099742; doi:10.1111/jvim.16958)
Supplement: Supplementary file 1 — Data S1. Supporting Information. [file JVIM-38-1765-s002.pdf]

Supplementary Materials Item 1: Search Strategy

PubMed

Concept 1: Viremia

"Viremia"[Mesh] OR "Viremia"[tw] OR "Viremias"[tw] OR viraemia[tw] OR viraemias[tw] OR viremic[tw] OR viraemic[tw]

Concept 2:EHV1

"Herpesvirus 1, Equid"[Mesh] OR “equine herpesvirus 1”[tw] OR “equine herpes virus 1”[tw] OR “Equine abortion Virus”[tw] OR “Equine abortion Viruses”[tw] OR “EHV 1”[tw] OR EHV1[tw] OR “equid herpesvirus 1”[tw] OR "equid herpesvirus type 1"[tw] OR "Equine herpesvirus myeloencephalopathy"[tw] OR “Equine herpes myeloencephalopathy”[tw] OR EHM[tw] OR "equine herpesvirus type 1"[tw] OR "equine herpes virus type 1"[tw] OR "alphaherpesvirus"[tw]

Concept 3: Horses

horses[mesh] OR horse[tw] OR horses[tw] OR equid*[tw] OR equine*[tw] OR equus[tw]

horses[mesh] OR horse[tw] OR horses[tw] OR equid*[tw] OR equine*[tw] OR equus[tw] OR Foals[tw] OR foal[tw] OR ponies[tw] OR pony[tw] OR mares[tw] OR mare[tw] OR gelding[tw] OR geldings[tw] OR stallion[tw] OR stallions[tw] OR donkey[tw] OR donkeys[tw] OR zebra[tw] OR zebras[tw] OR mule[tw] OR mules[tw]

Web of Science

Concept 1: Viremia

TS=("Viremia" OR "Viremias" OR viraemia OR viraemias OR viremic OR viraemic)

Concept 2:EHV1

TS=("equine herpesvirus 1" OR "equine herpes virus 1" OR "Equine abortion Virus" OR "Equine abortion Viruses" OR "EHV 1" OR "EHV1" OR "equid herpesvirus 1" OR "equid herpesvirus type 1" OR "Equine herpesvirus myeloencephalopathy" OR "Equine herpes myeloencephalopathy" OR "EHM" OR "equine herpesvirus type 1" OR "equine herpes virus type 1" OR "equid alphaherpesvirus 1" OR "Equine alphaherpesvirus 1")

Concept 3: Horses

TS=("horse" OR "horses" OR "equid" OR "equine" OR "equus")

Cochrane

AGRICOLA

Global Health

Cab Abstracts

Concept 1: Viremia

("Viremia" OR "Viremias" OR viraemia OR viraemias OR viremic OR viraemic)

AND

("equine herpesvirus 1" OR "equine herpes virus 1" OR "Equine abortion Virus" OR "Equine abortion Viruses" OR "EHV 1" OR "EHV1" OR "equid herpesvirus 1" OR "equid herpesvirus type 1" OR "Equine herpesvirus myeloencephalopathy" OR "Equine herpes myeloencephalopathy" OR "EHM" OR "equine herpesvirus type 1" OR "equine herpes virus type 1" OR "equid alphaherpesvirus 1" OR "Equine alphaherpesvirus 1")

AND

("horse" OR "horses" OR "equid" OR "equine" OR "equus")
