# Supplementary material for: Viremia and nasal shedding for the diagnosis of equine herpesvirus‐1 infection in domesticated horses
Source: J Vet Intern Med. 2023 Dec 9;38(3):1765–91. doi: 10.1111/jvim.16958 (PMC11099742; doi:10.1111/jvim.16958)
Supplement: Supplementary file 2 — Data S2. Supporting Information. [file JVIM-38-1765-s001.pdf]

Supplemental File 2. Excluded Studies.

Excluded study	Reason
Abdel-Rady A, El-Rahim I Abd, Abd El-Hameed S Gad El-Rab, Malek, SS. Clinical and molecular epidemiological study on herpesviruses infection among equid populations in upper Egypt. J Hellenic Vet Med Soc 2022;73(4):4861-4872.	Wrong study design
Allen GP. Risk factors for development of neurologic disease after experimental exposure to equine herpesvirus-1 in horses. Am J Vet Res. 2008 Dec;69(12):1595-600.	Wrong outcomes
Allen GP. Development of a real-time polymerase chain reaction assay for rapid diagnosis of neuropathogenic strains of equine herpesvirus-1. J Vet Diagn Invest. 2007 Jan;19(1):69-72.	Wrong outcomes
Allen GP, Breathnach CC. Quantification by real-time PCR of the magnitude and duration of leucocyte-associated viraemia in horses infected with neuropathogenic vs. non-neuropathogenic strains of EHV-1. Equine Vet J. 2006 May;38(3):252-7.	Wrong outcomes
Allen GP, Bryans JT. Molecular epizootiology, pathogenesis, and prophylaxis of equine herpesvirus-1 infections. Prog Vet Microbiol Immunol. 1986; 2:78-144.	Not original research
Allen GP. Antemortem detection of latent infection with neuropathogenic strains of equine herpesvirus-1 in horses. Am J Vet Res. 2006 Aug;67(8):1401-5.	Wrong outcomes
Ataseven VS, Dağalp SB, Güzel M, et al. Prevalence of equine herpesvirus-1 and equine herpesvirus-4 infections in equidae species in Turkey as determined by ELISA and multiplex nested PCR. Res Vet Sci. 2009 Apr;86(2):339-44.	Duplicate
Attili AR, Colognato R, Preziuso S, et al. Evaluation of three different vaccination protocols against EHV1/EHV4 infection in mares: Double Blind, randomized clinical trial. Vaccines (Basel). 2020 Jun 1;8(2):268.	Wrong study design
Azmi M. Field HJ, Rixon F, McLauchlan J. Protective immune responses induced by non-infectious L-particles of equine herpesvirus type-1: Implication of cellular immunity. J Microbiol 2002;40(1):11-19.	Wrong species
Balasuriya UB, Lee PA, Tsai YL, et al. Translation of a laboratory-validated equine herpesvirus-1 specific real-time PCR assay into an insulated isothermal polymerase chain reaction (iiPCR) assay for point-of-need diagnosis using POCKIT™ nucleic acid analyzer. J Virol Methods. 2017 Mar; 241:58-63.	Wrong outcomes
Banbura M, Chmielewska A, Tucholska A, Malicki K. Polymerase chain reaction in diagnosis of equine herpesvirus-1 miscarriage. Medycyna Weterynaryjna 2008 54(11):772-774.	Wrong outcomes
Banbura M, Chmielewska A, Tucholska A, Malicki K. Occurrence of equine herpes virus type-1 (EHV-1) - specific DNA sequences in peripheral blood leukocytes of horses. Medycyna Weterynaryjna 2000 56(8):521-523.	Wrong study design
Banbura M, Witkowski L, Chmielewska A, et al. Polymerase chain reaction in diagnosis of equine herpesvirus-1 miscarriage. Isolation of equine herpes viruses type 1 and 2 (EHV-1 and EHV-2) from foals infected with Rhodocaccus equi. Medycyna Weterynaryjna 2004;60(12):1333-1336.	Wrong study design
Banbura MW, Witkowski L, Chiviielewska A, Tucholska A. Mixed infections of equine herpes virus types 1 and 2 (EHV-1 and EHV-2). Medycyna Weterynaryjna Sep 2006;62(9):1071-1072	Wrong comparator
Bannai H, Kambayashi Y, Tsujimura K, et al. Persistence of virus-neutralizing antibodies in horses inoculated with two doses of a live equine herpesvirus type 1 vaccine with different vaccination intervals. J Equine Sci. 2021;32(3):99-102.	Wrong outcomes
Bannai H, Takahashi Y, Ohmura H, et al. Decreased virus-neutralizing antibodies against equine herpesvirus type 1 In nasal secretions of horses after 12-hour transportation. J Equine Vet Sci. 2021 Aug; 103:103665.	Wrong study design
Barrandeguy ME, Lascombes F, Llorente J, et al. High case-rate Equine herpesvirus-1 abortion outbreak in vaccinated polo mares in Argentina. Equine Vet Educ Jun 2002;14(3):132-135	Wrong study design
Batra SK, Jain NC, Tiwari SC. Isolation and characterization of EHV-1 herpesvirus associated with paralysis in equines. Ind J Anim Sci 1982;52(8):671-677	Wrong outcomes
Belák S. Molecular diagnosis of viral diseases, present trends and future aspects A view from the OIE Collaborating Centre for the application of polymerase chain reaction methods for diagnosis of viral diseases in veterinary medicine. Vaccine. 2007 Jul 26;25(30):5444-52.	Not original research
Bell SA, Balasuriya UB, Gardner IA, et al. Temporal detection of equine herpesvirus infections of a cohort of mares and their foals. Vet Microbiol. 2006 Sep 10;116(4):249-57.	Wrong outcomes
Berger S. Neuromuscular diseases in horses: a retrospective study from 1997-2007. Part 2: equine herpesvirus-myeloencephalopathy, equine motor neuron disease, equine degenerative myeloencephalopathy. Wiener Tierarztliche Monatsschrift 2009;96(3-4):87-95	Wrong outcomes

Bergmann V, Dietz O, Gangel H, et al. Clinical aspects and pathomorphology of equine paresis. Monatshefte Fur Veterinarmedizin 1990;45(9):310	Wrong outcomes
Bernhardt, D. In vitro diagnoses of equine herpesviruses (EHV1 and EHV4). Tierarztliche Umschau Feb 1 1993;48(2):67-72	Wrong outcomes
Berrios P, Maldonado R, Celedon MO, Cortes F. Equine rhinopneumonitis – isolation of the equine herpesvirus Type 1 from an outbreak of abortion in the IX region of Chile. Archivos De Medicina Veterinaria 1985;17(2):121-123	Wrong outcomes
Bilge DS, Babaoglu AR, Ataseven VS, et al. Determination of presence of equid alpha and gammaherpesvirus infections in foals with respiratory distress. Ankara Universitesi Veteriner Fakultesi Dergisi 2018 2018;65(1):63-68	Wrong study design
Borchers K, Slater J. A nested PCR for the detection and differentiation of EHV-1 and EHV-4. J Virol Methods. 1993 Dec 31;45(3):331-6.	Wrong outcomes
Borchers K, Steinbach F, Ludwig H. The importance of equine herpesvirus infections and their relevance for equine reproduction. Intl J Thymology 1993 1993;1(1):53-56	Wrong outcomes
Breathnach CC, Yeargan MR, Timoney JF, Allen GP. Detection of equine herpesvirus-specific effector and memory cytotoxic immunity in the equine upper respiratory tract. Vet Immunol Immunopathol. 2006 May 15;111(1-2):117-25.	Duplicate
Bridges CG, Edington N. Innate immunity during Equid herpesvirus 1 (EHV-1) infection. Clin Exp Immunol. 1986 Jul;65(1):172-81.	Wrong outcomes
Brosnahan MM, Erb HN, Perkins GA, et al. Serum iron parameters and acute experimental EHV-1 infection in horses. J Vet Intern Med. 2012 Sep-Oct;26(5):1232-5.	Wrong outcomes
Buchner HHF, Mostl K. Outbreak of an equine herpesvirus infection (EHV-1) in an university clinic. Wiener Tierarztliche Monatsschrift 1998 1998;85(3):87-93	Not in English
Bumgardner MK, Dutta SK, Campbell DL, Myrup AC. Lymphocytes from ponies experimentally infected with equine herpesvirus 1: subpopulation dynamics and their response to mitogens. Am J Vet Res. 1982 Jul;43(7):1308-10.	Wrong outcomes
Burgess BA, Tokateloff N, Manning S, et al. Nasal shedding of equine herpesvirus-1 from horses in an outbreak of equine herpes myeloencephalopathy in Western Canada. J Vet Intern Med. 2012 Mar-Apr;26(2):384-92.	Wrong outcomes
Bürki F, Nowotny N, Oulehla J, et al. Attempts to immunoprotect adult horses, specifically pregnant mares, with commercial vaccines against clinical disease induced by equine herpesvirus-1. Zentralbl Veterinarmed B. 1991 Aug;38(6):432-40.	Wrong outcomes
Burki F, Nowotny N, Rossmanith W, et al. Training of the immune system of foals against ERP virus infections by frequent vaccination with presently available commercial vaccines]. Dtsch Tierarztl Wochenschr Apr 1989;96(4):162-5	Not in English
Bürki F, Rossmanith W, Nowotny N, Pallan C, Möstl K, Lussy H. Viraemia and abortions are not prevented by two commercial equine herpesvirus-1 vaccines after experimental challenge of horses. Vet Q. 1990 Apr;12(2):80-6.	Duplicate
Burrell MH, Whitwell KE, Wood JL, Mumford JA. Pyrexia associated with respiratory disease in young thoroughbred horses. Vet Rec. 1994 Feb 26;134(9):219-20.	Wrong outcomes
Burrows R, Goodridge D. Studies of persistent and latent equid herpesvirus 1 and herpesvirus 3 infections in the Pirbright pony herd. Current topics in veterinary medicine and animal science 1984; 307-319	Wrong outcomes
Carlson JK, Traub-Dargatz JL, Lunn DP, et al. Equine viral respiratory pathogen surveillance at horse shows and sales. J Equ Vet Sci. 2013; 33(4): 229-237.	Wrong study design
Carvalho R, Passos LMF, Gouvea AMG, et al. Uso de um sistema de ELISA para detecçäo de anticorpos contra herpesvírus eqüino 1 (HVE-1) em éguas gestantes assintomáticas e potros recém-nascidos. Arq Bras Med Vet. zootec 2000/06 2000;52(3):200-7	Not original research
Choi SK, Kim JH, Gil-Jae C. A diagnosis of equine herpesvirus type 1 (EHV-1) myeloencephalopathy using real-time PCR. Korean J Vet Service 2014 2014;37(1):59-65	Not in English
Chong YC, Duffus WP. Immune responses of specific pathogen free foals to EHV-1 infection. Vet Microbiol. 1992 Oct;32(3-4):215-28.	Wrong outcomes
Chvala S, Nowotny N, Kotzab E, et al. Use of the meridian test for the detection of equine herpesvirus type 1 infection in horses with decreased performance. J Am Vet Med Assoc. 2004 Aug 15;225(4):554-9.	Wrong outcomes
Coignoul FL, Bertram TA, Cheville NF. Pathogenicity of equine herpesvirus 1 subtype 2 for foals and adult pony mares. Vet Microbiol. 1984 Oct;9(6):533-42.	Wrong outcomes
Coignoul FL, Bertram TA, Cheville NF. Functional and ultrastructural changes in neutrophils from mares and foals experimentally inoculated with a respiratory tract strain of equine herpesvirus-1. Am J Vet Res. 1984 Oct;45(10):1972-5.	Wrong outcomes

Coombs DK, Patton T, Kohler AK, Soboll G, Breathnach C, Townsend HG, Lunn DP. Cytokine responses to EHV-1 infection in immune and non-immune ponies. Vet Immunol Immunopathol. 2006 May 15;111(1-2):109-16.	Duplicate
Coombs DK, Patton T, Kohler AK, Soboll G, Breathnach C, Townsend HG, Lunn DP. Cytokine responses to EHV-1 infection in immune and non-immune ponies. Vet Immunol Immunopathol. 2006 May 15;111(1-2):109-16.	Wrong outcomes
Couetil L, Ivester K, Barnum S, Pusterla N. Equine respiratory viruses, airway inflammation and performance in thoroughbred racehorses. Vet Microbiol. 2021 Jun; 257:109070.	Wrong outcomes
Crandell RA, Mock RE, Lock TF. Vaccination of pregnant ponies against equine rhinopneumonitis. Am J Vet Res. 1980 Jul;41(7):994-6.	Wrong outcomes
Crowhurst FA, Dickinson G, Burrows R. An outbreak of paresis in mares and geldings associated with equid herpesvirus 1. Vet Rec. 1981 Dec 12;109(24):527-8.	Wrong study design
Diallo IS, Hewitson G, Wright LL, et al. Multiplex real-time PCR for the detection and differentiation of equid herpesvirus 1 (EHV-1) and equid herpesvirus 4 (EHV-4). Vet Microbiol. 2007 Jul 20;123(1-3):93-103.	Wrong outcomes
Doubli-Bounoua N, Richard EA, Léon A, et al. Multiple molecular detection of respiratory viruses and associated signs of airway inflammation in racehorses. Virol J. 2016 Nov 29;13(1):197.	Wrong outcomes
Drummer HE, Reynolds A, Studdert MJ, et al. Application of an equine herpesvirus 1 (EHV1) type-specific ELISA to the management of an outbreak of EHV1 abortion. Vet Rec. 1995 Jun 10;136(23):579-81.	Wrong outcomes
Dunowska M, Wilks CR, Studdert MJ, Meers J. Viruses associated with outbreaks of equine respiratory disease in New Zealand. N Z Vet J. 2002 Aug;50(4):132-9.	Wrong outcomes
Dunowska M, Wilks CR, Studdert MJ, Meers J. Equine respiratory viruses in foals in New Zealand. N Z Vet J. 2002 Aug;50(4):140-7.	Wrong outcomes
Dutta SK, Myrup A. Cell population dynamics virus existence and immune and interferon responses of lymphocytes in experimental equine herpesvirus 1 infection. Abstracts of the Annual Meeting of the American Society for Microbiology 1981;81():236-236	Meeting abstract only
Dutta SK, Myrup A, Bumgardner MK. Lymphocyte responses to virus and mitogen in ponies during experimental infection with equine herpesvirus 1. Am J Vet Res. 1980 Dec;41(12):2066-8.	Wrong outcomes
Dzieciatkowski T, Przybylski M, Chmielewska A, et al. Evaluation of a real-time PCR assay using the LightCycler (R) system for detection of equine herpesvirus type 1 DNA. Medycyna Weterynaryjna Jul 2008;64(7):918-921	Wrong outcomes
Edington N, Bridges CG. One way protection between equid herpesvirus 1 and 4 in vivo. Res Vet Sci 1990; 48:235-239.	Duplicate
Edington N, Bridges CG, Huckle A. Experimental reactivation of equid herpesvirus 1 (EHV 1) following the administration of corticosteroids. Equine Vet J. 1985 Sep;17(5):369-72.	Wrong outcomes
Edington N, Bridges CG, Griffiths L. Equine interferons following exposure to equid herpesvirus-1 or -4. J Interferon Res. 1989 Aug;9(4):389-92.	Wrong outcomes
Edington N, Bridges CG, Patel JR. Endothelial cell infection and thrombosis in paralysis caused by equid herpesvirus-1: equine stroke. Arch Virol. 1986;90(1-2):111-24.	Wrong outcomes
El-Husseini DM, Helmy NM, Tammam RH. Application of gold nanoparticle-assisted PCR for equine herpesvirus 1 diagnosis in field samples. Arch Virol. 2017 Aug;162(8):2297-2303.	Wrong study design
El-Husseini DM, Helmy NM, Tammam RH. The effect of gold nanoparticles on the diagnostic polymerase chain reaction technique for equine herpes virus 1 (EHV-1). Rsc Advances 2016 2016;6(60):54898-54903	Wrong outcomes
Erpenstein C, Siebert M, Failing K, Herbst W. Occurrence of nosocomial viral infections among stationary patients with different vaccinations in an equine hospital. Tierarztliche Praxis Ausgabe Grosstiere Nutztiere 2002;30(1):41-+	Wrong outcomes
Fitzpatrick DR, Studdert MJ. Immunologic relationships between equine herpesvirus type 1 (equine abortion virus) and type 4 (equine rhinopneumonitis virus). Am J Vet Res. 1984 Oct;45(10):1947-52.	Wrong outcomes
Foote CE, Love DN, Gilkerson JR, Whalley JM. Detection of EHV-1 and EHV-4 DNA in unweaned Thoroughbred foals from vaccinated mares on a large stud farm. Equine Vet J. 2004 May;36(4):341-5.	Wrong outcomes
Foote CE, Raidal SL, Pecenpetelovska G, et al. Inoculation of mares and very young foals with EHV-1 glycoproteins D and B reduces virus shedding following respiratory challenge with EHV-1. Vet Immunol Immunopathol. 2006 May 15;111(1-2):97-108.	Duplicate
Franz M, Goodman LB, Van de Walle GR, et al. A point mutation in a herpesvirus co-determines neuropathogenicity and viral shedding. Viruses. 2017 Jan 10;9(1):6.	Not original research

Friday PA, Scarratt WK, Elvinger F, Timoney PJ, Bonda A. Ataxia and paresis with equine herpesvirus type 1 infection in a herd of riding school horses. J Vet Intern Med. 2000 Mar-Apr;14(2):197-201.	Wrong outcomes
Frymus T, Kita J, Woyciechowska S, Ganowicz M. Foetal and neonatal foal losses on equine herpesvirus type 1(EHV-1) infected farms before and after EHV-1 vaccination was introduced. Pol Arch Weter. 1986;26(3-4):7-14.	Wrong outcomes
Garré B, Gryspeerdt A, Croubels S, De Backer P, Nauwynck H. Evaluation of orally administered valacyclovir in experimentally EHV1-infected ponies. Vet Microbiol. 2009 Mar 30;135(3-4):214-21.	Duplicate
Ghoniem SM, El Deeb AH, Aggour MG, Hussein HA. Development and evaluation of a multiplex reverse-transcription real-time PCR assay for detection of equine respiratory disease viruses. J Vet Diagn Invest. 2018 Nov;30(6):924-928.	Wrong outcomes
Gilkerson JR, Love DN, Whalley JM. Incidence of equine herpesvirus 1 infection in thoroughbred weanlings on two stud farms. Aust Vet J. 2000 Apr;78(4):277-8.	Wrong outcomes
Gilkerson JR, Whalley JM, Drummer HE, Studdert MJ, Love DN. Epidemiology of EHV-1 and EHV-4 in the mare and foal populations on a Hunter Valley stud farm: are mares the source of EHV-1 for unweaned foals. Vet Microbiol. 1999 Aug 16;68(1-2):27-34.	Wrong outcomes
Goehring LS, Soboll Hussey G, Gomez Diez M, et al. Plasma D-dimer concentrations during experimental EHV-1 infection of horses. J Vet Intern Med. 2013 Nov-Dec;27(6):1535-42.	Only viremia
Goehring LS, Landolt GA, Morley PS. Detection and management of an outbreak of equine herpesvirus type 1 infection and associated neurological disease in a veterinary teaching hospital. J Vet Intern Med. 2010 Sep-Oct;24(5):1176-83.	Wrong outcomes
Goehring LS, van Winden SC, van Maanen C, Sloet van Oldruitenborgh-Oosterbaan MM. Equine herpesvirus type 1-associated myeloencephalopathy in The Netherlands: a four-year retrospective study (1999-2003). J Vet Intern Med. 2006 May-Jun;20(3):601-7.	Wrong study design
Goodman LB, Wagner B, Flaminio MJ, et al. Comparison of the efficacy of inactivated combination and modified-live virus vaccines against challenge infection with neuropathogenic equine herpesvirus type 1 (EHV-1). Vaccine. 2006 Apr 24;24(17):3636-45.	Wrong outcomes
Gradzki Z, Boguta L. Use of PCR and cell culture method in the diagnosis and differentiation of EHV1 and EHV4 infections in horses. Medycyna Weterynaryjna Aug 2009;65(8):546-551.	Wrong outcomes
Greenwood RE, Simson AR. Clinical report of a paralytic syndrome affecting stallions, mares and foals on a thoroughbred studfarm. Equine Vet J. 1980 Jul;12(3):113-7.	Wrong outcomes
Gryspeerdt A, Vandekerckhove A, Van Doorsselaere J, et al. Description of an unusually large outbreak of nervous system disorders caused by equine herpesvirus 1 (EHV1) in 2009 in Belgium. Vlaams Diergeneeskundig Tijdschrift Mar-Apr 2011;80(2):147-153	Wrong outcomes
Gryspeerdt AC, Vandekerckhove AP, Garré B, Barbé F, Van de Walle GR, Nauwynck HJ. Differences in replication kinetics and cell tropism between neurovirulent and non-neurovirulent EHV1 strains during the acute phase of infection in horses. Vet Microbiol. 2010 May 19;142(3-4):242-53.	Duplicate
Taktaz Hafshejani T, Nekoei S, Vazirian B, Doosti A, Khamesipour F, Anyanwu MU. Molecular detection of equine herpesvirus types 1 and 4 infection in healthy horses in Isfahan Central and Shahrekord Southwest Regions, Iran. Biomed Res Int. 2015; 2015:917854.	Wrong study design
Henninger RW, Reed SM, Saville WJ, et al. Outbreak of neurologic disease caused by equine herpesvirus-1 at a university equestrian center. J Vet Intern Med. 2007 Jan-Feb;21(1):157-65.	Wrong study design
Holmes MA, Townsend HG, Kohler AK, et al. Immune responses to commercial equine vaccines against equine herpesvirus-1, equine influenza virus, eastern equine encephalomyelitis, and tetanus. Vet Immunol Immunopathol. 2006 May 15;111(1-2):67-80.	Wrong outcomes
Holmes MA, Townsend HG, Kohler AK, et al. Immune responses to commercial equine vaccines against equine herpesvirus-1, equine influenza virus, eastern equine encephalomyelitis, and tetanus. Vet Immunol Immunopathol. 2006 May 15;111(1-2):67-80.	Duplicate
Holz CL, Sledge DG, Kiupel M, et al. Histopathologic findings following experimental equine herpesvirus 1 Infection of horses. Front Vet Sci. 2019 Mar 4;6:59.	Wrong outcomes
Hu Z, Zhu C, Chang H, et al. Development of a single-tube duplex EvaGreen real-time PCR for the detection and identification of EHV-1 and EHV-4. Appl Microbiol Biotechnol. 2014 May;98(9):4179-86.	Wrong outcomes
Hussey GS, Goehring LS, Lunn DP, et al. Experimental infection with equine herpesvirus type 1 (EHV-1) induces chorioretinal lesions. Vet Res. 2013 Dec 5;44(1):118.	Duplicate
Soboll Hussey G, Hussey SB, Wagner B, Horohov DW, Van de Walle GR, Osterrieder N, Goehring LS, Rao S, Lunn DP. Evaluation of immune responses following infection of ponies with an EHV-1 ORF1/2 deletion mutant. Vet Res. 2011 Feb 7;42(1):23.	Duplicate

Irwin VL, Traub-Dargatz JL, Newton JR, Scase TJ, Davis-Poynter NJ, Nugent J, Creis L, Leaman TR, Smith KC. Investigation and management of an outbreak of abortion related to equine herpesvirus type 1 in unvaccinated ponies. Vet Rec. 2007 Mar 17;160(11):378-80.	Wrong study design
Jelocnik M, Nyari S, Anstey S, et al. Real-time fluorometric and end-point colorimetric isothermal assays for detection of equine pathogens C. psittaci and equine herpes virus 1: validation, comparison and application at the point of care. BMC Vet Res. 2021 Aug 19;17(1):279.	Wrong outcomes
Kalad MA, Hanafy M, Warda SA, et al. Prospective studies of equine herpes virus-1 Myeloencephalopathy in Egypt 2012. Ippologia Sep-Dec 2013;24(3-4):25-31	Wrong study design
Kraft W, Fiebiger I, Grabner, A. EHV-1-myeloencephalitis des Pferdes. Berl Munch Tierarztl Wochenschr 1982;95(17):321-325	Wrong outcomes
Kraft W, Grabner A, Fiebiger I. EHV-1 induced myeloencephalitis in horses. Berl Munch Tierarztl Wochenschr 1982;95(17):321-325	Wrong outcomes
Krogsrud J. Infeksjon med hesteherpesvirus type 1. Norsk veterinaertidsskrift 1983;95(4):243-244.	Not original research
Kydd JH, Hannant D, Mumford JA. Residence and recruitment of leucocytes to the equine lung after EHV-1 infection. Vet Immunol Immunopathol. 1996 Jun 15;52(1-2):15-26.	Wrong outcomes
Kydd JH, Hannant D, Robinson RS, et al. Vaccination of foals with a modified live, equid herpesvirus-1 gM deletion mutant (RacHΔgM) confers partial protection against infection. Vaccine. 2020 Jan 10;38(2):388-398.	Duplicate
Kydd JH, Hannant D, Robinson RS, et al. Vaccination of foals with a modified live, equid herpesvirus-1 gM deletion mutant (RacHΔgM) confers partial protection against infection. Vaccine. 2020 Jan 10;38(2):388-398.	Duplicate
Lechmann J, Schoster A, Ernstberger M, et al. A novel PCR protocol for detection and differentiation of neuropathogenic and non-neuropathogenic equid alphaherpesvirus 1. J Vet Diagn Invest. 2019 Sep;31(5):696-703.	Wrong outcomes
van Maanen C, Willink DL, Smeenk LA, et al. An equine herpesvirus 1 (EHV1) abortion storm at a riding school. Vet Q. 2000 Apr;22(2):83-7.	Wrong outcomes
van Maanen C, Sloet van Oldruitenborgh-Oosterbaan MM, Damen EA, Derksen AG. Neurological disease associated with EHV-1-infection in a riding school: clinical and virological characteristics. Equine Vet J. 2001 Mar;33(2):191-6.	Wrong outcomes
Mackie JT, MacLeod GA, Reubel GH, Studdert MJ. Diagnosis of equine herpesvirus 1 abortion using polymerase chain reaction. Aust Vet J. 1996 Nov;74(5):390-1.	Wrong outcomes
Mapes S, Leutenegger CM, Pusterla N. Nucleic acid extraction methods for detection of EHV-1 from blood and nasopharyngeal secretions. Vet Rec. 2008 Jun 28;162(26):857-9.	Wrong study design
Mason DK, Luk CM, Watkins KL. Absolute monocyte counts as an aid to early diagnosis of equine herpesvirus 1 (EHV1) infection in Hong Kong thoroughbred horses. J Equine Vet Sci 1989;9(3):133-136	Wrong outcomes
Mason DK, Watkins KL, McNie JT, Luk CM. Haematological measurements as an aid to early diagnosis and prognosis of respiratory viral infections in thoroughbred horses. Vet Rec. 1990 Apr 14;126(15):359-63.	Wrong outcomes
Matsumura T, Kondo T, Sugita S, et al. An equine herpesvirus type 1 recombinant with a deletion in the gE and gI genes is avirulent in young horses. Virology. 1998 Mar 1;242(1):68-79.	Wrong study design
Matsumura T, Sugiura T, Imagawa H, et al. Epizootiological aspects of type 1 and type 4 equine herpesvirus infections among horse populations. J Vet Med Sci. 1992 Apr;54(2):207-11.	Only nasal shedding
Matsumura T, Yokota S, Imagawa H, et al. Sero- and molecular-epizootiological studies on equine herpesvirus type 1 (EHV-1) infection among race horses: An occurrence of respiratory disease with nervous disorders. J Equine Sci 1994 1994;5(2):59-67	Wrong outcomes
McBrearty KA, Murray A, Dunowska M. A survey of respiratory viruses in New Zealand horses. N Z Vet J. 2013 Sep;61(5):254-61.	Wrong outcomes
McBrearty KA, Murray A, Dunowska M. A survey of respiratory viruses in New Zealand horses. N Z Vet J. 2013 Sep;61(5):254-61.	Duplicate
McCartan CG, Russell MM, Wood JL, Mumford JA. Clinical, serological and virological characteristics of an outbreak of paresis and neonatal foal disease due to equine herpesvirus-1 on a stud farm. Vet Rec. 1995 Jan 7;136(1):7-12.	Wrong outcomes
McCulloch J, Williamson SA, Powis SJ, Edington N. The effect of EHV-1 infection upon circulating leucocyte populations in the natural equine host. Vet Microbiol. 1993 Oct;37(1-2):147-61.	Wrong outcomes

van der Meulen K, Caij B, Pensaert M, Nauwynck H. Absence of viral envelope proteins in equine herpesvirus 1-infected blood mononuclear cells during cell-associated viremia. Vet Microbiol. 2006 Mar 31;113(3-4):265-73.	Duplicate
Mumford EL, Traub-Dargatz JL, Salman MD, Collins JK, Getzy DM, Carman J. Monitoring and detection of acute viral respiratory tract disease in horses. J Am Vet Med Assoc. 1998 Aug 1;213(3):385-90.	Wrong outcomes
Mumford JA, Bates J. Trials of an inactivated equid herpesvirus 1 vaccine: challenge with a subtype 2 virus. Vet Rec. 1984 Apr 14;114(15):375-81.	Wrong outcomes
Mumford JA, Rossdale PD, Jessett DM, et al. Serological and virological investigations of an equid herpesvirus 1 (EHV-1) abortion storm on a stud farm in 1985. J Reprod Fertil Suppl. 1987; 35:509-18.	Wrong outcomes
Muscat KE, Padalino B, Hartley CA, et al. Equine transport and changes in equid herpesvirus' status. Front Vet Sci. 2018 Sep 25; 5:224.	Wrong outcomes
Nemoto M, Ohta M, Tsujimura K, et al. Direct detection of equine herpesvirus type 1 DNA in nasal swabs by loop-mediated isothermal amplification (LAMP). J Vet Med Sci. 2011 Sep;73(9):1225-7.	Wrong outcomes
Nemoto M, Tsujimura K, Yamanaka T, et al. Loop-mediated isothermal amplification assays for detection of Equid herpesvirus 1 and 4 and differentiating a gene-deleted candidate vaccine strain from wild-type Equid herpesvirus 1 strains. J Vet Diagn Invest. 2010 Jan;22(1):30-6.	Wrong outcomes
Newton JR, Wood JL, Chanter N. A case control study of factors and infections associated with clinically apparent respiratory disease in UK Thoroughbred racehorses. Prev Vet Med. 2003 Jul 30;60(1):107-32.	Wrong outcomes
Oldruitenborgh-Oosterbaan MMS, van; Binkhorst G.J. Neurologische afwijkingen ten gevolge van equine herpesvirus type 1 en neuritis caudae equinae bij het paard (een literatuuroverzicht). Tijdschr Diergeneeskd 1984;109(24):1027-1035	Not original research
Ons E, Van Brussel L, Lane S, et al. Efficacy of a Parapoxvirus ovis-based immunomodulator against equine herpesvirus type 1 and Streptococcus equi equi infections in horses. Vet Microbiol. 2014 Oct 10;173(3-4):232-40.	Only viremia
Osterrieder N, Neubauer A, Brandmuller C, Kaaden OR. Efficacy of recombinant EHV1 vaccines. Tierarztliche Umschau Nov 1996;51(11):683-687	Wrong species
Paillot R, Daly JM, Juillard V, et al. Equine interferon gamma synthesis in lymphocytes after in vivo infection and in vitro stimulation with EHV-1. Vaccine. 2005 Aug 22;23(36):4541-51.	Wrong outcomes
Paillot R, Daly JM, Luce R, et al. Frequency and phenotype of EHV-1 specific, IFN-gamma synthesising lymphocytes in ponies: the effects of age, pregnancy and infection. Dev Comp Immunol. 2007;31(2):202-14.	Wrong outcomes
Paillot R, Ellis SA, Daly JM, et al. Characterisation of CTL and IFN-gamma synthesis in ponies following vaccination with a NYVAC-based construct coding for EHV-1 immediate early gene, followed by challenge infection. Vaccine. 2006 Mar 6;24(10):1490-500.	Wrong study design
Passamonti F, Lepri E, Marenzoni ML, et al. Equid herpesvirus type 1 (EHV-1) congenital infection: An outbreak on thoroughbred stud farm. Ippologia Dec 2006;17(4):21-25	Wrong outcomes
Patel JR, Bateman H, Williams J, Didlick S. Derivation and characterisation of a live equid herpes virus-1 (EHV-1) vaccine to protect against abortion and respiratory disease due to EHV-1. Vet Microbiol. 2003 Jan 2;91(1):23-39.	Duplicate
Perkins GA, Goodman LB, Dubovi EJ, et al. Detection of equine herpesvirus-1 in nasal swabs of horses by quantitative real-time PCR. J Vet Intern Med. 2008 Sep-Oct;22(5):1234-8.	Wrong outcomes
Perl S, Haines D, Yakobson B, et al. Paresis in horses associated with equine herpes virus 1 infection. Israel J Vet Med ;52(4):132-136	Meeting abstract only
Price D, Barnum S, Mize J, Pusterla N. Investigation of the use of non-invasive samples for the molecular detection of EHV-1 in horses with and without clinical infection. Pathogens. 2022 May 13;11(5):574.	Wrong outcomes
Pusterla N, Mapes S, Wilson WD. Diagnostic sensitivity of nasopharyngeal and nasal swabs for the molecular detection of EHV-1. Vet Rec. 2008 Apr 19;162(16):520-1.	Wrong comparator
Pronost S, Legrand L, Pitel PH, et al. Outbreak of equine herpesvirus myeloencephalopathy in France: a clinical and molecular investigation. Transbound Emerg Dis. 2012 Jun;59(3):256-63.	Duplicate
Pusterla N, Hussey SB, Mapes S, et al. Molecular investigation of the viral kinetics of equine herpesvirus-1 in blood and nasal secretions of horses after corticosteroid-induced recrudescence of latent infection. J Vet Intern Med. 2010 Sep-Oct;24(5):1153-7.	Wrong study design

Pusterla N, Kass PH, Mapes S, et al. Surveillance programme for important equine infectious respiratory pathogens in the USA. Vet Rec. 2011 Jul 2;169(1):12.	Wrong study design
Pusterla N, Mapes S, Madigan JE, et al. Prevalence of EHV-1 in adult horses transported over long distances. Vet Rec. 2009 Oct 17;165(16):473-5.	Duplicate
Pusterla N, Mapes S, Wademan C, et al. Investigation of the role of lesser characterised respiratory viruses associated with upper respiratory tract infections in horses. Vet Rec. 2013 Mar 23;172(12):315.	Wrong outcomes
Pusterla N, Hussey SB, Mapes S, et al. Comparison of four methods to quantify Equid herpesvirus 1 load by real-time polymerase chain reaction in nasal secretions of experimentally and naturally infected horses. J Vet Diagn Invest. 2009 Nov;21(6):836-40.	Wrong outcomes
Pusterla N, Wilson WD, Mapes S, et al. Characterization of viral loads, strain and state of equine herpesvirus-1 using real-time PCR in horses following natural exposure at a racetrack in California. Vet J. 2009 Feb;179(2):230-9.	Duplicate
Pusterla N, Wilson WD, Mapes S. Comparison of the Diagnostic Sensitivity of Nasopharyngeal and Nasal Swabs and Use of Viral Loads for the Molecular Diagnosis of Equine Herpesvirus-1 Infection. American Association of Equine Practitioners. Proceedings of the ... annual convention 2007;(53):220-224	Not peer reviewed
Rosch X, Rosch B, Engel M. Infection of equines with the EHV-1 and EHV-4 virus. Praktische Tierarzt Nov 1 1992;73(11):1050-&	Not original research
Rusvai M, Kucsera L, Palfi V. Prevalence of equine viral diseases in Hungary, the main aspects of their control. Magyar Allatorvosok Lapja Aug 1996;51(8):499-503	Wrong outcomes
Schnabel CL, Wimer CL, Perkins G, et al. Deletion of the ORF2 gene of the neuropathogenic equine herpesvirus type 1 strain Ab4 reduces virulence while maintaining strong immunogenicity. BMC Vet Res. 2018 Aug 22;14(1):245.	Wrong outcomes
Schroer U, Lange A, Glatzel P, et al. The relevance of equine herpesvirus 1 (EHV-1) infection in a German thoroughbred stud: vaccination, abortion and diagnostics. Berl Munch Tierarztl Wochenschr Feb 2000;113(2):53-59	Not in English
Schroer U, Lange A, Glatzel P, et al. The relevance of equine herpesvirus 1 (EHV-1) infection in a German thoroughbred stud: vaccination, abortion and diagnostics. Berl Munch Tierarztl Wochenschr Feb 2000;113(2):53-59	Duplicate
Schulman M, Becker A, Ganswindt S, et al. The effect of consignment to broodmare sales on physiological stress measured by faecal glucocorticoid metabolites in pregnant Thoroughbred mares. BMC Vet Res. 2014 Jan 17; 10:25.	Duplicate
Schulman M, Becker A, Ganswindt S, et al. The effect of consignment to broodmare sales on physiological stress measured by faecal glucocorticoid metabolites in pregnant Thoroughbred mares. BMC Vet Res. 2014 Jan 17; 10:25.	Wrong outcomes
Scott JC, Dutta SK, Myrup AC. In vivo harboring of equine herpesvirus-1 in leukocyte populations and subpopulations and their quantitation from experimentally infected ponies. Am J Vet Res. 1983 Jul;44(7):1344-8.	Wrong outcomes
Seo MG, Ouh IO, Lee SK, et al. Molecular detection and genetic characteristics of equine herpesvirus in Korea. Pathogens. 2020 Feb 11;9(2):110.	Wrong study design
Sharma PC, Cullinane AA, Onions DE, Nicolson L. Diagnosis of equid herpesviruses -1 and -4 by polymerase chain reaction. Equine Vet J. 1992 Jan;24(1):20-5.	Wrong outcomes
Sinclair R, Mumford JA. Rapid detection of equine herpesvirus type-1 antigens in nasal swab specimens using an antigen capture enzyme-linked immunosorbent assay. J Virol Methods. 1992 Sep;39(3):299-310.	Wrong outcomes
Singh BK. Immunological response of ponies to an inactivated-Indian strain of equine herpes virus-1. Indian J Anim Sci Oct 2002;72(10):831-834	Wrong outcomes
Singh BK, Tandon SN, Virmani N. Immune responses to inactivated oil adjuvanted Equine Herpes Virus-1 using different emulsifiers in horses. Ind J Biotech Jan 2006;5(1):42-46	Wrong outcomes
Slater JD, Gibson JS, Barnett KC, Field HJ. Chorioretinopathy associated with neuropathology following infection with equine herpesvirus-1. Vet Rec. 1992 Sep 12;131(11):237-9.	Wrong outcomes
Slater JD, Gibson JS, Field HJ. Pathogenicity of a thymidine kinase-deficient mutant of equine herpesvirus 1 in mice and specific pathogen-free foals. J Gen Virol. 1993 May;74 (Pt 5):819-28.	Duplicate
Smith FL, Watson JL, Spier SJ, et al. Frequency of shedding of respiratory pathogens in horses recently imported to the United States. J Vet Intern Med. 2018 Jul;32(4):1436-1441.	Wrong outcomes
Smith KC, Whitwell KE, Binns MM, et al. Abortion of virologically negative foetuses following experimental challenge of pregnant pony mares with equid herpesvirus 1. Equine Vet J. 1992 Jul;24(4):256-9.	Wrong outcomes

Smith KC, Whitwell KE, Mumford JA, et al. An immunohistological study of the uterus of mares following experimental infection by equid herpesvirus 1. Equine Vet J. 1993 Jan;25(1):36-40.	Wrong outcomes
Smith KC, Whitwell KE, Binns MM, et al. Abortion of virologically negative foetuses following experimental challenge of pregnant pony mares with equid herpesvirus 1. Equine Vet J. 1992 Jul;24(4):256-9.	Only viremia
Smith KL, Li Y, Breheny P, Cook RF, et al. New real-time PCR assay using allelic discrimination for detection and differentiation of equine herpesvirus-1 strains with A2254 and G2254 polymorphisms. J Clin Microbiol. 2012 Jun;50(6):1981-8.	Wrong outcomes
Soboll G, Hussey SB, Whalley JM, et al. Antibody and cellular immune responses following DNA vaccination and EHV-1 infection of ponies. Vet Immunol Immunopathol. 2006 May 15;111(1-2):81-95.	Duplicate
Soboll G, Breathnach CC, Kydd JH, et al. Vaccination of ponies with the IE gene of EHV-1 in a recombinant modified live vaccinia vector protects against clinical and virological disease. Vet Immunol Immunopathol. 2010 May 15;135(1-2):108-117.	Duplicate
Sonis JM, Goehring LS. Nasal shedding of equid herpesvirus type 1 and type 4 in hospitalized, febrile horses. J Equine Vet Sci Sep 2013;33(9):756-759.	Wrong study design
Strang C, Newton R. Control and disease clearance after neurological EHV-1 in the UK. Vet Rec. 2017 Dec 23;181(25):678-679.	Wrong outcomes
Sugiura T, Matsumura T, Fukunaga Y. Isolation and identification of viruses from racehorses with pyrexia. Bulletin of Equine Research Institute 1989 1989;(26):53-5	Not original research
Sutton GA, Viel L, Carman PS, Boag BL. Pathogenesis and clinical signs of equine herpesvirus-1 in experimentally infected ponies in vivo. Can J Vet Res. 1998 Jan;62(1):49-55.	Wrong outcomes
Tewari SC, Sharma PC, Prasad S. Abortions associated with equine herpes virus-1 in army and civil establishments in northern parts of India. Ind J Anim Sci Jul 1987;57(7):623-627	Wrong outcomes
Thein P. Infection of the central nervous system of horses with equine herpesvirus serotype 1. J S Afr Vet Assoc. 1981 Sep;52(3):239-41.	Wrong outcomes
Theunissen GT, van Essen GJ, van Maanen C, Schrijver RS. Field trial with a subunit rhinopneumovaccine. Tijdschr Diergeneeskd Feb 1 1995;120(3):72-4	Wrong outcomes
Tsujimura Koji, Shiose T, Kokubun A, et al. A study on an inoculum dose of equine herpes virus type 1 (EHV-1) mutant defective in the open reading frame of glycoprotein E and its vaccine effects. J Equine Sci March 2004;15(1):22-22	Meeting abstract only
Turan N, Yildirim F, Altan E, et al. Molecular and pathological investigations of EHV-1 and EHV-4 infections in horses in Turkey. Res Vet Sci. 2012 Dec;93(3):1504-7.	Wrong outcomes
Van de Walle GR, Goupil R, Wishon C, Damiani A, Perkins GA, Osterrieder N. A single-nucleotide polymorphism in a herpesvirus DNA polymerase is sufficient to cause lethal neurological disease. J Infect Dis. 2009 Jul 1;200(1):20-5.	Wrong outcomes
van den Wollenberg L, van Dijck w, van Oldruitenborgh-Oosterbaan MMS, van Maanen K. A horse with neurological rhinopneumonia; how long to isolate? Tijdschr Diergeneeskd Apr 1 2017;142(4):30-35.	Not original research
van der Meulen KM, Nauwynck HJ, Pensaert MB. Equine herpesvirus type 1 abortion, neonatal foal death and nervous system disorders diagnosed in Belgium in 1999. Vlaams Diergeneeskundig Tijdschrift Jan-Feb 2000;69(1):38-41.	Wrong outcomes
van der Meulen K, Vercauteren G, Nauwynck H, Pensaert M. A local epidemic of equine herpesvirus 1-induced neurological disorders in Belgium. Vlaams Diergeneeskundig Tijdschrift Sep-Oct 2003;72(5):366-372.	Wrong study design
Varshney JP, Singh BK, Gupta AK, Uppal PK. Investigations in Kathiawari horses suffering from upper respiratory tract infection. Ind Vet J Aug 1993;70(8):710-712	Wrong outcomes
Velloso Alvarez A, Jose-Cunilleras E, Dorrego-Rodriguez A, et al. Detection of equine herpesvirus-1 (EHV-1) in urine samples during outbreaks of equine herpesvirus myeloencephalopathy. Equine Vet J. 2023 Sep 12. doi: 10.1111/evj.14007.	Wrong outcomes
Virmani N, Verma PC, Panisup AS, et al. Comparative studies on neurotropic properties of indigenous strains of equine herpes Virus-1. Ind J Anim Sci Apr 2005;75(4):393-396.	Wrong species
Wang L, Raidal SL, Pizzirani A, Wilcox GE. Detection of respiratory herpesviruses in foals and adult horses determined by nested multiplex PCR. Vet Microbiol. 2007 Mar 31;121(1-2):18-28.	Duplicate
Weiblen R, Rabuske M, Rebelatto MC, et al. Abortion due to equine herpesvirus in southern Brazil. Braz J Med Biol Res. 1994 Jun;27(6):1317-20.	Wrong outcomes

Welch HM, Bridges CG, Lyon AM, et al. Latent equid herpesviruses 1 and 4: detection and distinction using the polymerase chain reaction and co-cultivation from lymphoid tissues. J Gen Virol. 1992 Feb;73 (Pt 2):261-8.	Duplicate
Welch HM, Bridges CG, Lyon AM, et al. Latent equid herpesviruses 1 and 4: detection and distinction using the polymerase chain reaction and co-cultivation from lymphoid tissues. J Gen Virol. 1992 Feb;73 (Pt 2):261-8.	Wrong outcomes
Wilson ME, Holz CL, Kopec AK, et al. Coagulation parameters following equine herpesvirus type 1 infection in horses. Equine Vet J. 2019 Jan;51(1):102-107.	Wrong outcomes
Wilsterman S, Soboll-Hussey G, Lunn DP, et al. Equine herpesvirus-1 infected peripheral blood mononuclear cell subpopulations during viremia. Vet Microbiol. 2011 Apr 21;149(1-2):40-7.	Wrong outcomes
Wilsterman S, Soboll-Hussey G, Lunn DP, et al. Equine herpesvirus-1 infected peripheral blood mononuclear cell subpopulations during viremia. Vet Microbiol. 2011 Apr 21;149(1-2):40-7.	Duplicate
Wintzer HJ, vd Bossche G, Ludwig H, Bischof B. Seuchenverlauf nach EHV-1-Infektion in einem Reitpferdebestand [Infectious course of equine herpesvirus 1 infection in a riding stable]. Dtsch Tierarztl Wochenschr. 1987 Mar 9;94(3):149-52.	Wrong outcomes
Wintzer HJ, vd Bossche G, Ludwig H, Bischof B. Seuchenverlauf nach EHV-1-Infektion in einem Reitpferdebestand [Infectious course of equine herpesvirus 1 infection in a riding stable]. Dtsch Tierarztl Wochenschr. 1987 Mar 9;94(3):149-52.	Duplicate
Witherspoon DM. Vaccination against equine herpesvirus 1 and equine influenza infection. Vet Rec. 1984 Oct 6;115(14):363.	Wrong outcomes
Yactor J, Kasper KS, Kohler AK, et al. Detection of nasal shedding of EHV-1 & 4 at equine show events and sales by multiplex real-time PCR. American Association of Equine Practitioners. Proceedings of the annual convention 2006;52():223-227	Wrong study design
Yanni MI, Ebtsam AA, Ali HA, Hanna NM. Verification of molecular and conventional techniques used in the diagnosis of equine herpes virus in some Egyptian governorates. J Appl Vet Sci 2021;6(1):1-8.	Wrong study design
Yilmaz H, Altan E, Turan N, et al. First report on the frequency and molecular detection of neuropathogenic EHV-1 in Turkey. J Equine Vet Sci 2012;32(9):525-530	Wrong outcomes
Zarski LM, Giessler KS, Jacob SI, et al. Identification of Host Factors Associated with the Development of Equine Herpesvirus Myeloencephalopathy by Transcriptomic Analysis of Peripheral Blood Mononuclear Cells from Horses. Viruses. 2021 Feb 24;13(3):356.	Wrong outcomes
Zarski LM, Vaala WE, Barnett DC, et al. A live-attenuated equine influenza Vaccine Stimulates innate immunity in equine respiratory epithelial cell cultures That could provide protection from equine Herpesvirus 1. Front Vet Sci. 2021 Jun 10; 8:674850.	Wrong outcomes
